# Zeolite-Based Composite Nanomaterials for Organic Micropollutant Removal: Structure–Property–Performance Relationships and Practical Challenges

**DOI:** 10.3390/nano16100635

**Published:** 2026-05-20

**Authors:** Nurlybayeva Aisha, Sarova Nurbanu, Ainur Seitkan, Rakhmetullayeva Raikhan, Myrzabek Yermakhanov, Tazhkenova Gaukhar, Matniyazova Gulsim, Zhanbulatova Gaukhar, Nurlybayev Olzhas, Rustem Ergali

**Affiliations:** 1 Department of Chemistry and Chemical Technology, Faculty of Technology, M.Kh. Dulaty Taraz Regional University, Taraz 080000, Kazakhstan; rustem_ergali@mail.ru (N.A.); nur.olzhas_91@mail.ru (N.O.); 2 Department of Pharmaceutical Sciences, Faculty of Chemistry and Biology, Abai Kazakh National Pedagogical University, Almaty 050010, Kazakhstan; 3 Higher School of Natural Sciences, Astana International University, Astana 010000, Kazakhstan; seitkanainur.77@mail.ru (A.S.); gulsim.matniyazova@mail.ru (M.G.); 4 Department of Chemistry and Technology of Organic Substances, Natural Compounds and Polymers, Al-Farabi Kazakh National University, Almaty 050040, Kazakhstan; raikhan.rakhmetullayeva@gmail.com; 5 Department of Chemistry, Mukhtar Auezov South Kazakhstan University, Shymkent 160012, Kazakhstan; myrza1964@mail.ru (M.Y.); rustem-ergali79@mail.ru (R.E.); 6 Department of Chemistry, Faculty of Natural Sciences, L.N. Gumilyov Eurasian National University, Astana 010000, Kazakhstan; 7 Centre for Educational Programmes, Autonomous Educational Organization “Nazarbayev Intellectual Schools”, Astana 010000, Kazakhstan; gaukhar.zhanbulatova20@gmail.com

**Keywords:** zeolite-based nanocomposites, composite nanomaterials, organic micropollutants, adsorption mechanisms, PFAS removal, photocatalytic degradation, water treatment, nanostructured adsorbents, π–π stacking, micropore confinement

## Abstract

Zeolite-based composite nanomaterials represent a versatile and mechanistically rich platform for the removal of organic micropollutants (OMPs)—including pharmaceuticals, endocrine-disrupting compounds, pesticides, and per- and polyfluoroalkyl substances (PFAS)—from contaminated water systems. Although pristine zeolite frameworks provide well-defined microporous architectures, tunable Si/Al ratios, and ion-exchange capacity, their intrinsic hydrophilicity restricts interaction diversity and limits performance toward the structurally heterogeneous OMPs prevalent in real aquatic environments. Composite integration with carbonaceous nanophases, functional polymers and surfactants, and catalytically active metal oxide nanoparticles substantially extends this interaction repertoire, yielding multifunctional materials whose adsorption performance exceeds that of the individual components. Drawing on a systematic survey of peer-reviewed literature published between 2016 and 2026, this review develops a mechanism-oriented, structure–property–performance framework examining five dominant adsorption mechanisms—electrostatic attraction, π–π stacking, hydrogen bonding, hydrophobic partitioning, and micropore confinement—in relation to composite nanoarchitecture, surface chemistry, and structural parameters. The modulating influence of realistic water matrix conditions on adsorption efficiency is critically assessed, alongside challenges of regeneration, long-term stability, metal leaching, and the persistent gap between laboratory-scale synthesis and scalable deployment. Priority research directions are identified, including standardized performance evaluation under environmentally representative conditions and rational design of hierarchical multifunctional nanocomposites from earth-abundant and waste-derived precursors.

## 1. Introduction

The widespread occurrence of trace organic contaminants in natural water bodies and treated effluents represents one of the most pressing challenges in contemporary environmental science. Organic micropollutants (OMPs)—a structurally heterogeneous group encompassing pharmaceuticals, personal care products (PPCPs), endocrine-disrupting compounds (EDCs), pesticides, and per- and polyfluoroalkyl substances (PFAS)—are continuously discharged into aquatic systems through municipal wastewater effluents, hospital discharges, agricultural runoff, and diffuse urban sources. Environmental concentrations typically range from ng L^−1^ to low µg L^−1^; however, the biological activity of many of these compounds at sub-therapeutic doses, combined with their resistance to conventional degradation pathways, renders them of considerable ecotoxicological and public health concern [1,2,3]. Chronic low-level exposure to OMP mixtures has been shown to disrupt endocrine function, impair reproductive success in aquatic organisms, and promote the selection of antimicrobial resistance in microbial communities [4,5,6]. These impacts directly undermine the objectives of SDG 6 (Clean Water and Sanitation) and SDG 3 (Good Health and Well-Being), reinforcing the urgency of developing effective and scalable removal technologies.

Conventional water and wastewater treatment infrastructure was not designed for trace organic removal, and OMP elimination efficiencies in full-scale plants are highly variable and strongly compound-dependent, with many pharmaceuticals passing through biological treatment largely unaltered [7,8]. Advanced oxidation processes, nanofiltration, and reverse osmosis can achieve substantially higher removal rates, but their adoption is constrained by energy demand, operational complexity, and the generation of concentrated waste streams [9]. Among available polishing strategies, adsorption remains particularly attractive for its operational simplicity, adaptability to existing infrastructure, and capacity for selective contaminant capture without generating transformation products of uncertain toxicity.

Activated carbon—in powdered and granular forms—has long been the benchmark adsorbent for OMP removal [10]. Its performance is, however, sensitive to competitive adsorption by natural organic matter (NOM), pore fouling, and variability in surface chemistry across source materials and activation conditions. Regeneration limitations further constrain cost-effectiveness when treating dilute, multicomponent contaminant streams [11]. These shortcomings have motivated extensive research into alternative porous materials offering tunable adsorption selectivity, chemically defined active surfaces, and improved regeneration characteristics.

Among inorganic porous materials, zeolites occupy a distinctive position. Their crystalline aluminosilicate frameworks define a precise and reproducible micropore architecture, impart a permanent negative framework charge enabling cation exchange, and confer exceptional thermal and chemical stability [12,13]. The Si/Al ratio provides a fundamental handle for tuning surface hydrophobicity, and the structural diversity of zeolite topologies—spanning large-pore FAU and BEA frameworks, medium-pore MFI, and the naturally abundant clinoptilolite—offers a broad palette for adsorption engineering. Nevertheless, the intrinsic hydrophilicity of aluminosilicate surfaces limits the affinity of unmodified zeolites for neutral and weakly polar organic molecules, which constitute a large fraction of environmentally relevant OMPs [14]. Moreover, pure ion-exchange mechanisms are selective for cationic species, while many pharmaceuticals and EDCs are non-ionic or exist across multiple speciation states over the pH range of natural waters [15].

These recognized shortcomings have driven a sustained effort to develop composite materials that retain the structural advantages of zeolite frameworks while extending their interaction capacity through integration with secondary functional phases. Carbonaceous materials—activated carbon, graphene oxide, biochar, and carbon nanotubes—introduce aromatic π-electron domains capable of engaging in π–π stacking and hydrophobic partitioning with aromatic contaminants. Polymer and surfactant modification create organophilic surface environments that alter the partitioning behavior of hydrophobic OMPs while simultaneously addressing processability constraints. Functionalization with metal oxides, particularly TiO_2_, ZnO, and iron species, opens the additional dimension of catalytic degradation, enabling hybrid materials to combine adsorption-driven contaminant concentration with subsequent photocatalytic or Fenton-type mineralization [16,17].

A critical and often underappreciated challenge concerns the gap between laboratory-measured adsorption capacities and performance achievable under realistic water matrix conditions. Most published studies employ single-solute systems in deionized water, conditions that systematically overestimate removal efficiencies by eliminating competitive adsorption by NOM, electrostatic shielding by dissolved ions, and pore fouling [18]. When evaluated in natural water or secondary wastewater effluent matrices, reported capacities frequently decline substantially, and mechanistic interpretation becomes considerably more complex due to the coexistence of multiple competing species and the potential for surface modification by humic substances [19].

**Figure 1:**
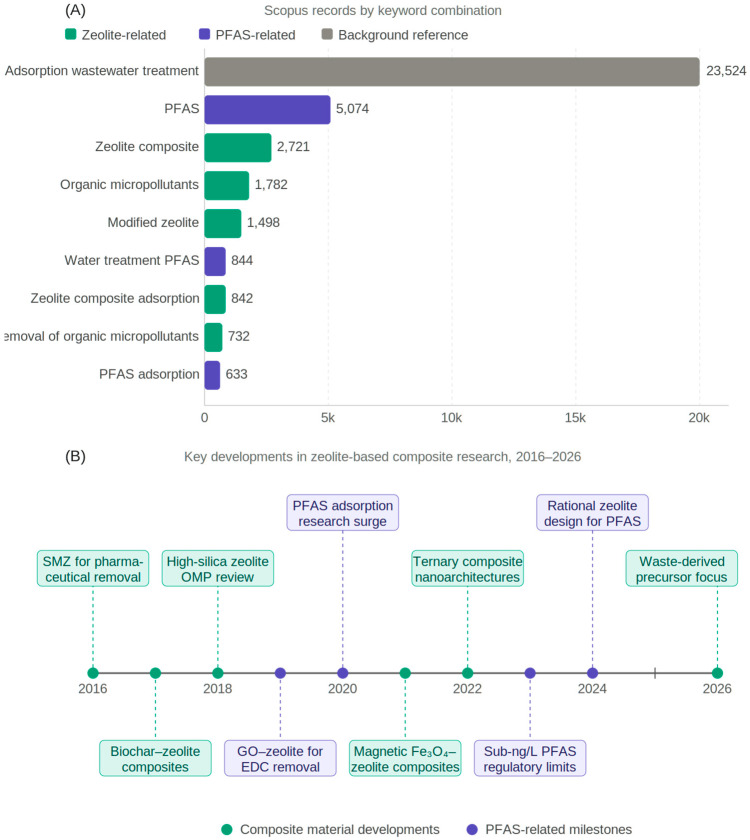
Bibliometric overview of the systematic literature search and key research milestones. (**A**) Distribution of Scopus records retrieved using five keyword combinations related to zeolite-based composite adsorbents and organic micropollutant removal. The background reference query (“adsorption wastewater treatment”; n = 23,524) is shown in grey; PFAS-specific queries are shown in purple; zeolite-related queries are shown in green. (**B**) Selected milestones in the development of zeolite-based composite nanomaterials for OMP removal, 2016–2026. Green markers denote advances in composite material design; purple markers denote PFAS-related regulatory and research developments.

The present review is based on a systematic literature search conducted in the Scopus database covering the publication period January 2016 to March 2026. The search was restricted to peer-reviewed journal articles and review articles published in English; conference proceedings, book chapters, and grey literature were excluded. Primary keyword combinations employed in the search included: “zeolite composite” AND “adsorption”, “modified zeolite” AND “organic micropollutants”, “zeolite” AND “PFAS” AND “adsorption”, “zeolite composite” AND “wastewater treatment”, and “zeolite” AND “photocatalysis” AND “micropollutants”. The distribution of retrieved records across the five keyword combinations is illustrated in Figure 1A, and key milestones in the development of zeolite-based composite materials for OMP removal over the review period are summarized in Figure 1B.

Initial screening was conducted at the title and abstract level, applying the following exclusion criteria: studies focused exclusively on inorganic contaminants (heavy metals, ammonium, nitrate) without reporting OMP removal data; studies employing model dye contaminants (methylene blue, Congo Red, and analogues) as the sole performance benchmark without reporting data for pharmaceuticals, pesticides, EDCs, or PFAS; studies reporting synthesis or characterization of zeolite-based materials without adsorption or degradation performance data; and studies conducted under conditions bearing no plausible relationship to water treatment applications (e.g., non-aqueous systems, extreme temperature or pressure). Full-text assessment was subsequently applied to all records passing initial screening, with final inclusion requiring explicit reporting of adsorption capacity or removal efficiency data, identification of at least one dominant adsorption mechanism, and sufficient characterization of experimental conditions (pH, initial contaminant concentration, sorbent dosage, water matrix) to permit mechanistic interpretation.

The combined keyword search retrieved 5793 records. Following removal of duplicates (n = 614), 5179 records were screened at title and abstract level, of which 4683 were excluded because of the criteria described above. The remaining 496 records underwent full-text review; 399 were subsequently excluded due to insufficient mechanistic content, inadequate experimental characterization, or exclusive focus on inorganic contaminants. Backward and forward citation tracking was applied to all records meeting inclusion criteria, enabling identification of foundational studies predating the 2016 search window and emerging research directions not yet captured by keyword-based retrieval; studies identified through this process were subject to the same inclusion and exclusion criteria as database-retrieved records. The final corpus comprised approximately 96 peer-reviewed primary studies, supplemented by authoritative reviews and reference texts cited where synthesis of broader context was required. The complete study selection procedure conforms to the PRISMA 2020 reporting framework for systematic reviews. Where quantitative trends are discussed, data are drawn from studies reporting results under comparable experimental conditions; where direct comparison is not possible due to methodological heterogeneity, this limitation is explicitly noted.

As illustrated in Figure 1A, the Scopus search retrieved between 633 and 2721 documents for keyword combinations directly combining zeolite-related terms with OMP removal or adsorption processes. The query “zeolite composite adsorption” yielded 842 documents out of 23,524 records retrieved for the broader “adsorption wastewater treatment” query, indicating that zeolite-composite-specific literature, while substantial, remains a focused subset of the wider adsorption research landscape. The relatively modest share of zeolite composite studies within the total adsorption literature—approximately 3.6%—underscores the need for dedicated mechanistic analysis, particularly given that comparable searches for carbon-based composites and modified clay minerals (e.g., bentonites) return substantially larger document sets, pointing to an uneven distribution of research effort across adsorbent classes. PFAS-specific queries—“PFAS” (5074 records) and “water treatment PFAS” (844 records)—reflect the rapidly growing regulatory and scientific interest in this recalcitrant contaminant class, for which zeolite-based composites represent a mechanistically promising yet systematically underexplored removal platform. The temporal distribution of key research milestones illustrated in Figure 1B reveals a clear acceleration in composite material diversity from 2019 onward, driven by the emergence of GO–zeolite systems for EDC removal, ternary nanoarchitectures, and PFAS-targeted composite designs coinciding with tightening regulatory limits.

The present review addresses this need by providing a mechanism-oriented, structure–property–performance analysis of zeolite-based composite nanomaterials for OMP removal, drawing on the systematic literature corpus described above. The review is structured as follows: Section 2 examines OMP physicochemical descriptors governing adsorption behavior; Section 3 evaluates the structural basis and performance boundaries of pristine zeolite frameworks and the three principal composite classes; Section 4 analyses dominant adsorption mechanisms and the influence of structural parameters; Section 5 addresses water matrix and operational effects; and Section 6 and Section 7 discuss regeneration, stability, and future research priorities.

## 2. Organic Micropollutants in Aquatic Systems: Classification, Occurrence, and Physicochemical Basis of Adsorption

### 2.1. Chemical Classification and Environmental Occurrence

The term “organic micropollutants” encompasses a structurally and functionally heterogeneous group of biologically active trace compounds whose environmental concentrations—typically between 1 ng L^−1^ and several µg L^−1^—are orders of magnitude below those of classical regulated pollutants, yet sufficient to elicit measurable physiological responses in aquatic organisms [14,15]. Six principal chemical classes dominate the current monitoring and research literature: pharmaceuticals and their metabolites, personal care products (PPCPs), endocrine-disrupting compounds (EDCs), pesticides and their transformation products, industrial additives, and per- and polyfluoroalkyl substances (PFAS). Each class is characterized by distinct emission pathways, environmental persistence, and interaction behaviour with sorbent materials, which collectively determine the demands placed on any removal technology.

Pharmaceuticals constitute the most extensively monitored OMP class, driven by their continuous and geographically diffuse release through municipal wastewater effluents. Compounds such as the anticonvulsant carbamazepine, the anti-inflammatory diclofenac, the antibiotic sulfamethoxazole, and the analgesic ibuprofen are routinely detected in surface waters across Europe, Asia, and North America at concentrations of tens to hundreds of ng L^−1^ [16,17]. Carbamazepine has gained particular attention as a persistence indicator owing to its near-complete resistance to conventional biological treatment and its detection in groundwater and drinking water sources [18]. Antibiotics present an additional dimension of concern beyond their direct ecotoxicity: sub-inhibitory environmental concentrations exert selection pressure on microbial communities, contributing to the dissemination of antimicrobial resistance genes in aquatic systems.

EDCs—including natural and synthetic estrogens (17β-estradiol, 17α-ethinylestradiol), bisphenol A, and phthalate plasticisers—are capable of binding to hormonal receptors at concentrations as low as 1–10 ng L^−1^, inducing feminisation phenomena in fish populations and disrupting reproductive cycles in amphibians [19,20]. Their widespread detection in treated wastewater effluents reflects both their structural stability and the limited efficacy of activated sludge processes toward hydrophobic, slowly biodegradable compounds. Pesticides, introduced into aquatic systems predominantly through agricultural surface runoff, contribute a further layer of complexity through their high structural diversity, spanning chlorinated herbicides, organophosphate insecticides, and triazine fungicides with markedly different mobilities and persistence characteristics [21].

PFAS represent perhaps the most intractable subgroup within the OMP category. The exceptional strength of the carbon–fluorine bond (bond dissociation energy ~544 kJ mol^−1^) renders perfluorooctanoic acid (PFOA), perfluorooctane sulfonate (PFOS), and their short-chain analogues essentially inert to biological mineralisation under environmental conditions [22]. Concentrations in the low ng L^−1^ range are now routinely detected in surface waters, groundwater, and drinking water worldwide, including in remote polar regions [23]. The bioaccumulative and potentially carcinogenic properties of long-chain PFAS have prompted regulatory limits in the sub-ng L^−1^ range in several jurisdictions, setting extreme demands on any removal technology and making them a demanding test case for adsorption-based approaches.

The concept of pseudo-persistence is central to understanding why OMP contamination cannot be addressed through conventional environmental attenuation alone [24]. Unlike legacy persistent organic pollutants, many pharmaceuticals and PPCPs undergo partial photodegradation or biotransformation with half-lives of days to weeks. However, their continuous and uninterrupted anthropogenic input maintains steady-state aquatic concentrations that are effectively indistinguishable from those of compounds with inherent environmental persistence. This dynamic equilibrium between input and degradation means that even moderate removal efficiencies in wastewater treatment—50–70%, as commonly reported for many pharmaceuticals—are insufficient to prevent their accumulation in receiving water bodies [25,26].

A further complicating factor is the multicomponent nature of real contamination scenarios. Municipal wastewater effluents typically contain simultaneous mixtures of dozens of pharmaceuticals, PPCPs, and EDCs, each at trace concentrations [27]. Mixture toxicity effects—additive, synergistic, or antagonistic responses arising from co-exposure to structurally distinct compounds at individually sub-threshold concentrations—have been demonstrated for pharmaceutical mixtures in multiple aquatic species, reinforcing the inadequacy of single-compound risk assessments and treatment evaluations [28]. For adsorption-based technologies, multicomponent systems introduce competitive sorption effects that substantially complicate the mechanistic picture and often reduce effective capacity relative to single-solute laboratory measurements.

### 2.2. Physicochemical Descriptors Governing Adsorption Behaviour

The rational design of adsorbent materials for OMP removal requires a systematic understanding of the molecular descriptors that control interaction pathways between contaminant molecules and sorbent surfaces. Because OMPs span an exceptionally wide range of physicochemical properties, no single descriptor provides a universal predictor of adsorption affinity; rather, removal efficiency emerges from the interplay of several molecular characteristics operating simultaneously under the constraints imposed by water matrix composition.

Hydrophobicity, quantified as the octanol–water partition coefficient (log K_ow_), is the single most frequently applied descriptor in OMP adsorption studies and provides a first-order predictor of affinity toward nonpolar sorbent domains. Neutral, hydrophobic compounds with log K_ow_ > 3—including many steroid hormones, bisphenol A, and certain pesticides—exhibit strong partitioning from the aqueous phase into hydrophobic surface regions of carbon-modified zeolite composites and surfactant-functionalised frameworks, driven primarily by the thermodynamic penalty of maintaining a nonpolar molecule in an aqueous environment [29]. However, log K_ow_ alone fails as a predictor for ionisable compounds, whose effective hydrophobicity is pH-dependent and must be expressed through the distribution coefficient log D at a specified pH, and for PFAS, whose unique surfactant character creates adsorption affinities that do not scale predictably with conventional hydrophobicity metrics [30].

Acid–base speciation, governed by the compound’s dissociation constant (pKa), is the second critical controlling parameter for ionisable OMPs—most pharmaceuticals and many pesticides. The degree of ionization at a given solution pH determines both the net charge of the contaminant molecule and the nature of its electrostatic interaction with the sorbent surface. Diclofenac (pKa 4.0) exists predominantly as the diclofenac anion at neutral pH and thus interacts electrostatically with positively charged sorbent sites, while exhibiting repulsion from the negatively charged zeolite framework; conversely, cationic pharmaceuticals such as metformin (pKa 12.4) are protonated across the full environmental pH range and interact favorably with unmodified zeolite surfaces through ion exchange [31,32]. Compounds with multiple ionizable groups—including fluoroquinolone antibiotics, which carry both carboxyl and amine functionalities—exhibit pH-dependent speciation that can shift from cationic to zwitterionic to anionic across the pH range of 4–9, producing non-monotonic adsorption–pH relationships that are frequently misinterpreted in the absence of explicit speciation analysis [33].

Molecular geometry and size govern physical accessibility of adsorption sites within microporous frameworks. Effective molecular diameters of environmentally relevant OMPs range from approximately 0.5 nm for small pharmaceuticals such as metronidazole to over 1.2 nm for larger steroid hormones and macrolide antibiotics [34,35]. Given that zeolite pore apertures typically span 0.38–0.74 nm depending on framework topology (Table 1), steric exclusion from micropores can be a decisive factor limiting adsorption capacity for bulky molecules, even when thermodynamically favourable interactions exist at accessible surface sites. This size-selectivity can, however, be exploited deliberately: the molecular sieving properties of small-pore frameworks have been proposed as a mechanism for selective PFAS capture based on chain length discrimination [36].

Specific functional group interactions provide additional adsorption pathways beyond hydrophobicity and electrostatics. Aromatic ring systems present in many pharmaceuticals—including the phenyl and heterocyclic rings of carbamazepine, diclofenac, and fluoroquinolones—are capable of engaging in π–π stacking interactions with graphitic or aromatic domains introduced through carbon modification of zeolite composites [37,38]. Hydrogen bonding between electron-donating groups (–OH, –NH_2_, –C=O) on the contaminant and surface hydroxyl or amine functionalities of polymer-modified zeolites provides a further, often underestimated, contribution to adsorption affinity for polar pharmaceuticals [7] Surface complexation, relevant to metal-oxide-functionalised composites, enables specific coordination of compounds bearing carboxylate or phosphonate groups to Lewis acid sites on TiO_2_ or iron oxide surfaces [39].

**Figure 2:**
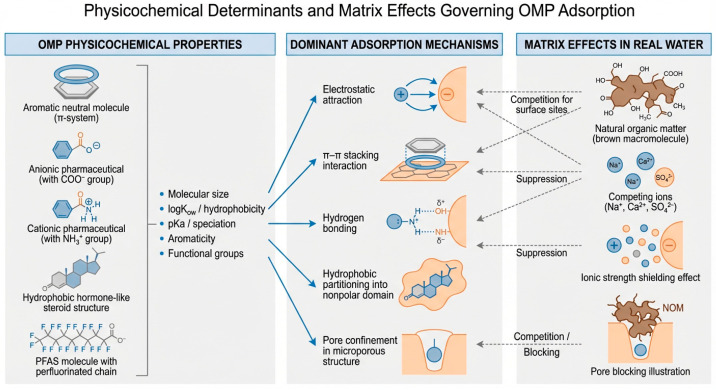
Conceptual frameworks illustrating the relationships between the physicochemical properties of organic micropollutants (**left**), the dominant adsorption mechanisms governing their interaction with zeolite-based composite sorbents (**centre**), and the principal water matrix effects modulating adsorption performance under realistic environmental conditions (**right**). NOM = natural organic matter.

The interplay among these descriptors, and their modulation by water matrix components—dissolved organic matter (DOM), inorganic ions, and pH—is illustrated conceptually in Figure 2. A representative selection of environmentally relevant OMPs and their key physicochemical descriptors relevant to adsorption behaviour is summarised in Table 1.

**Table 1:** Physicochemical properties of selected organic micropollutants relevant to adsorption on zeolite-based composite materials.

Compound	Class	log K_ow_	pKa	Charge at pH 7	Molecular Diameter (nm)	Key Adsorption Pathway	Reference
Carbamazepine	Pharmaceutical (anticonvulsant)	2.45	13.9	Neutral	~0.71	Hydrophobic partitioning, π–π stacking	[15,21]
Diclofenac	Pharmaceutical (anti-inflammatory)	4.51	4.0	Anionic	~0.76	Electrostatic attraction (modified surfaces), π–π stacking	[39,40]
Sulfamethoxazole	Pharmaceutical (antibiotic)	0.89	1.6; 5.7	Anionic	~0.72	Electrostatic interactions, hydrogen bonding	[20]
Ibuprofen	Pharmaceutical (analgesic/NSAID)	3.97	4.9	Anionic	~0.65	Hydrophobic partitioning, electrostatic attraction	[24,27]
17β-Estradiol	EDC (natural estrogen)	4.01	10.4	Neutral	~0.85	Hydrophobic partitioning, π–π stacking	[5,16]
17α-Ethinylestradiol	EDC (synthetic estrogen)	3.67	10.4	Neutral	~0.85	Hydrophobic partitioning, π–π stacking	[16,21]
Bisphenol A	EDC (industrial additive)	3.32	9.6; 10.2	Neutral	~0.70	Hydrophobic partitioning, π–π stacking	[20,22]
Atrazine	Pesticide (herbicide)	2.61	1.7	Neutral	~0.62	Hydrophobic partitioning, pore confinement	[21,25]
Ciprofloxacin	Pharmaceutical (fluoroquinolone antibiotic)	0.28	6.1; 8.7	Zwitterionic	~0.78	π–π stacking, electrostatic interactions	[38,39]
Metformin	Pharmaceutical (antidiabetic)	−1.43	12.4	Cationic	~0.55	Ion exchange, electrostatic attraction	[24,40]
Atenolol	Pharmaceutical (β-blocker)	0.16	9.6	Cationic	~0.74	Ion exchange, electrostatic attraction	[27,39]
PFOA	PFAS (perfluoroalkyl acid)	3.40	−0.5	Anionic	~0.58	Hydrophobic partitioning + electrostatic attraction (dual-mode)	[30,31]
PFOS	PFAS (perfluoroalkyl sulfonate)	4.49	−3.3	Anionic	~0.62	Hydrophobic partitioning + electrostatic attraction (dual-mode)	[5,30]

Note: logK_ow_ = octanol–water partition coefficient; pKa = acid dissociation constant; molecular diameters are approximate kinetic diameters estimated from crystallographic van der Waals radii or derived from molecular modelling (PM7 semiempirical method); values reflect the minimum projection diameter of the energy-minimised molecular geometry and are intended for qualitative comparison with zeolite pore aperture dimensions rather than precise steric exclusion predictions. Charge state given at pH 7.0. EDC = endocrine-disrupting compound; PFAS = per- and polyfluoroalkyl substances; NSAID = non-steroidal anti-inflammatory drug. References correspond to the article reference list.

The molecular diameter data compiled in Table 1 warrant brief methodological clarification. Kinetic diameters reported for OMP molecules are derived from crystallographic van der Waals dimensions or molecular modelling approaches and represent approximate minimum projection diameters of the energy-minimised molecular geometry [34,35]. These values are inherently method-dependent: diameters estimated from van der Waals radii typically exceed those derived from molecular dynamics simulations by 0.05–0.15 nm, and flexible molecules may adopt conformations with substantially different effective cross-sections depending on solvent environment and pH-dependent protonation state. In the context of zeolite adsorption, molecular diameters should therefore be interpreted as indicative rather than precise predictors of micropore accessibility: a molecule whose estimated diameter marginally exceeds a zeolite pore aperture may still diffuse into the channel system through conformational flexibility or framework breathing, while a molecule nominally smaller than the aperture may be sterically hindered by pendant functional groups oriented perpendicular to the diffusion axis. With these caveats in mind, the comparison of OMP molecular diameters with zeolite pore aperture dimensions provides a useful first-order framework for predicting size-exclusion effects and identifying contaminant–framework combinations where steric constraints are likely to limit adsorption capacity.

The data presented in Table 1 illustrate the breadth of physicochemical diversity that any zeolite-based composite adsorbent must accommodate. Log K_ow_ values span nearly four orders of magnitude—from sulfamethoxazole (0.89) to diclofenac (4.51)—while charge states at environmentally relevant pH 7 range from fully neutral (carbamazepine, bisphenol A, atrazine) to strongly anionic (diclofenac, PFOA, ibuprofen). This diversity has a direct and fundamental consequence: no single interaction mechanism can account for the removal of structurally dissimilar OMPs across a composite material, and the dominant adsorption pathway shifts substantially depending on both the molecular identity of the contaminant and the surface chemistry of the sorbent [40]. Neutral hydrophobic compounds such as carbamazepine are driven toward nonpolar sorbent domains through hydrophobic partitioning and π–π stacking [41], whereas anionic pharmaceuticals such as diclofenac require positively charged or metal-functionalised surface sites to achieve electrostatic uptake. PFAS present a distinct case: despite their formally anionic character at pH 7, their removal is governed by a combination of hydrophobic partitioning of the perfluorinated chain and electrostatic attraction of the charged head group, a dual-mode mechanism that places specific demands on composite architecture not met by any single-phase material [42].

Equally important is the recognition that these intrinsic molecular descriptors do not operate in isolation in real water systems. The presence of dissolved organic matter, competing inorganic ions, and pH shifts characteristic of natural and wastewater matrices can substantially suppress, redirect, or in some cases enhance the interaction pathways identified under idealised laboratory conditions. The complex interplay between OMP molecular properties, dominant adsorption mechanisms, and the modulating influence of water matrix components is summarised conceptually in Figure 2.

As illustrated in Figure 2, the removal of any given OMP by a zeolite-based composite is rarely governed by a single dominant mechanism. Rather, adsorption performance reflects the simultaneous and often competing contributions of multiple interaction pathways, whose relative importance is determined by the molecular structure of the contaminant, the surface chemistry and pore architecture of the composite material, and the physicochemical composition of the surrounding aqueous environment. This mechanistic complexity underpins the central analytical framework of the present review and motivates the structure–property–performance approach developed in the sections that follow.

## 3. Zeolite Frameworks as Adsorption Platforms: Structural Basis, Composite Engineering, and Performance Boundaries

### 3.1. Structural Characteristics of Zeolite Frameworks and Their Relevance to OMP Adsorption

Zeolites are crystalline aluminosilicates whose frameworks are constructed from corner-sharing SiO_4_ and AlO_4_ tetrahedra, generating ordered three-dimensional microporous architectures of precisely defined geometry. Isomorphic substitution of Si^4+^ by Al^3+^ introduces a permanent negative charge into the framework, balanced by exchangeable extra-framework cations—typically Na^+^, K^+^, Ca^2+^, or NH_4_^+^—residing within the channel systems. This charge-balancing mechanism underpins the ion-exchange capacity that has historically made zeolites effective materials for ammonium removal and heavy metal remediation in water treatment [15,43]. However, when the target contaminants shift from inorganic ions to structurally diverse organic micropollutants, the governing parameters become considerably more complex, and the limitations of unmodified zeolite frameworks become apparent.

Three structural parameters collectively determine zeolite adsorption behavior toward OMPs: framework topology and pore geometry, the Si/Al ratio, and the nature and accessibility of extra-framework cation sites.

Framework topology defines both the pore aperture dimensions and the channel connectivity, which together control the physical accessibility of internal adsorption sites to organic molecules of varying size and geometry. FAU-type frameworks (zeolite X and Y) present large super cages (~1.3 nm internal diameter) accessible through 12-membered ring windows of ~0.74 nm, facilitating diffusion of moderately sized pharmaceutical molecules including diclofenac (kinetic diameter ~0.76 nm) and carbamazepine (~0.71 nm). MFI-type zeolites (ZSM-5, silicalite-1) provide a three-dimensional intersecting channel system with 10-membered ring apertures of approximately 0.51 × 0.55 nm and 0.53 × 0.56 nm, offering shape-selective adsorption but imposing steric constraints on bulkier contaminants. BEA frameworks (Beta zeolite) combine 12-membered ring channels (~0.66–0.76 nm) in a three-dimensional arrangement that provides both high accessibility and large internal surface area. In contrast, one-dimensional channel systems such as mordenite (MOR, 12-membered ring, ~0.65 × 0.70 nm main channel) impose diffusion bottlenecks under dynamic flow conditions, since blocked pore mouths cannot be bypassed through alternative pathways [42]. The naturally abundant clinoptilolite (HEU topology), with its intersecting 10- and 8-membered ring channels, represents the most widely used natural zeolite in water treatment applications due to its availability and ion-exchange capacity, though its relatively narrow pore apertures restrict the diffusion of larger organic molecules [44].

The Si/Al ratio is the primary determinant of zeolite surface hydrophilicity and, consequently, of competitive adsorption between water molecules and organic contaminants for internal adsorption sites. Low-silica zeolites (Si/Al < 5, including zeolites A, X, and natural clinoptilolite) possess high framework charge density and strong affinity for polar molecules and water, which severely limits uptake of neutral organic contaminants through competitive exclusion. High-silica and pure-silica frameworks (Si/Al > 20, including high-silica ZSM-5, silicalite-1, and dealuminated zeolite Y) exhibit markedly reduced surface polarity and water affinity, creating an internal environment more conducive to hydrophobic partitioning of neutral pharmaceuticals and EDCs. Jiang et al. systematically demonstrated that high-silica zeolites outperform their low-silica counterparts in the adsorption of carbamazepine and other neutral pharmaceuticals from aqueous solution, attributing this to reduced competitive hydration within the micropore channels [15]. However, this advantage is compound-class-specific: for ionizable pharmaceuticals whose adsorption depends substantially on electrostatic interactions, the reduced framework charge density of high-silica zeolites may diminish rather than enhance removal efficiency.

Extra-framework cation sites contribute to OMP adsorption through Lewis’s acid–base interactions and, in some cases, through direct coordination with functional groups of the contaminant. Divalent cations such as Ca^2+^ and Mg^2+^ in ion-exchange positions can act as bridging species between the negatively charged zeolite framework and anionic organic contaminants, enhancing uptake under conditions where direct electrostatic adsorption would be unfavorable [45]. However, these same sites are also the primary targets of competition from dissolved inorganic cations in natural water matrices, which frequently reduces OMP uptake under realistic conditions.

Despite the structural precision and chemical stability that make zeolites attractive adsorption platforms, pristine frameworks exhibit two fundamental limitations when applied to OMP removal. First, the hydrophilic nature of aluminosilicate surfaces—even in high-silica variants—places zeolites at a disadvantage relative to activated carbon for the adsorption of neutral, hydrophobic contaminants: activated carbon presents extensive graphitic π-electron systems and heterogeneous surface chemistry enabling both strong hydrophobic partitioning and π–π interactions that zeolite micropores cannot replicate [45,46]. Second, the structural uniformity that confers precise size-selectivity simultaneously restricts the diversity of accessible interaction mechanisms: a given zeolite framework engages contaminant molecules through a limited subset of interaction pathways, whereas the removal of structurally diverse OMPs demands materials capable of engaging in electrostatic, hydrophobic, π–π, and hydrogen-bonding interactions simultaneously. These two limitations—insufficient hydrophobic character and restricted interaction diversity—define the boundaries of pristine zeolite performance and establish the motivation for composite material engineering.

### 3.2. Carbon–Zeolite Composite Materials

The integration of carbonaceous phases into zeolite frameworks represents the most extensively investigated composite strategy for enhancing OMP adsorption, and its rationale is mechanistically straightforward: carbonaceous materials introduce the aromatic π-electron domains and hydrophobic surface regions that are absent from aluminosilicate frameworks, while the zeolite component contributes structural stability, ion-exchange capacity, and size-selective micropore confinement. The resulting composites engage OMP molecules through a broader spectrum of interaction pathways than either component alone [46].

Activated carbon–zeolite composites exploit the complementarity between activated carbon’s high specific surface area (typically 800–1500 m^2^ g^−1^) and heterogeneous pore distribution, and the zeolite’s ordered micropore architecture and surface charge. Studies on the removal of carbamazepine and diclofenac from aqueous solution have demonstrated that physical mixing or co-granulation of the two components already produces synergistic adsorption behavior, with composite materials achieving higher equilibrium capacities than either component at equivalent mass fractions [45]. More sophisticated synthesis approaches—including in situ crystallization of zeolite phases within activated carbon matrices and chemical vapor deposition of carbonaceous coatings onto pre-formed zeolite particles—yield materials with improved interfacial contact between the two phases and more uniform distribution of interaction sites [47].

Biochar–zeolite composites have attracted increasing attention as potentially low-cost, sustainable alternatives derived from agricultural waste streams. Biochar introduces both hydrophobic aromatic domains and oxygen-containing functional groups (carboxyl, hydroxyl, carbonyl) that participate in hydrogen bonding with polar pharmaceuticals. Co-pyrolysis of biomass precursors with zeolite components or alkaline activation of biochar in the presence of zeolite-forming silica and alumina sources have been employed to produce composites in which both phases contribute distinct and complementary adsorption sites. Reported improvements in antibiotic and phenolic compound removal in multicomponent systems suggest that biochar domains enhance adsorption of hydrophobic contaminants while the zeolite component selectively retains cationic species through ion exchange [48,49].

Graphene oxide (GO) and reduced graphene oxide (rGO)–zeolite composites exploit the two-dimensional aromatic surface of graphene sheets as a platform for π–π stacking interactions with aromatic pharmaceutical molecules. GO–zeolite composites have demonstrated enhanced removal of bisphenol A and oestrogen compounds relative to the parent zeolite, attributed to the extended aromatic surface area provided by GO sheets anchored to the external zeolite surface [50,51]. The oxygen-containing functional groups of GO (epoxide, carboxyl, hydroxyl) additionally provide sites for hydrogen bonding and electrostatic interactions with polar contaminants, making GO–zeolite composites particularly versatile for structurally diverse OMP mixtures. Carbon nanotube (CNT)–zeolite hybrids similarly introduce high-aspect-ratio aromatic domains with strong π–π stacking capacity and have been reported to improve adsorption kinetics through enhanced external surface area and reduced intraparticle diffusion path lengths [52,53].

A critical observation emerging from the carbon–zeolite composite literature is that the magnitude of performance improvement relative to the parent zeolite is strongly dependent on synthesis route, carbon loading, and the specific contaminant–composite pairing, and is not universally observed across all OMP classes [13,50]. Excessive carbon loading can partially obstruct zeolite micropore entrances, reducing the contribution of the zeolite component and diminishing the synergistic benefit. Quantitative evidence from N_2_ adsorption studies indicates that this effect becomes significant at carbon loadings exceedingly approximately 15–20 wt% in activated carbon–zeolite systems and at GO contents above 5–10 wt% in graphene oxide–zeolite composites [52,54]. At these thresholds, BET surface area measurements reveal a disproportionate reduction in micropore volume relative to total surface area—a hallmark of pore mouth occlusion rather than uniform surface coverage—with micropore volumes declining by 30–60% relative to the parent zeolite at carbon loadings of 20–30 wt% [49,55]. The mechanistic basis of this occlusion differs between carbonaceous phase types: activated carbon particles physically block pore entrances through particle bridging at zeolite crystal boundaries, whereas graphene oxide sheets and carbon nanotubes—owing to their high aspect ratio and tendency to aggregate on external zeolite surfaces—form continuous overlying films that seal micropore apertures across multiple adjacent crystals [52,54]. This structural distinction has practical consequences for composite design: for activated carbon systems, occlusion can be partially mitigated by reducing carbon particle size to below the zeolite crystal dimensions; for GO and CNT systems, covalent functionalization of the carbon phase prior to composite assembly reduces aggregation tendency and produces more uniform surface distribution at equivalent mass loadings. Furthermore, the aromatic interaction pathways introduced by carbonaceous phases are most effective for contaminants with aromatic ring systems—pharmaceuticals such as carbamazepine, diclofenac, and fluoroquinolones—but provide limited enhancement for aliphatic compounds or highly polar molecules. Representative carbon–zeolite composites and their adsorption performance are summarized in Table 2.

Alongside these performance advantages, carbon–zeolite composites present a set of intrinsic limitations that are systematically underreported in the primary literature and warrant explicit critical assessment. Pore blockage at the carbon–zeolite interface represents the most structurally consequential limitation: deposition of carbonaceous phases—whether through impregnation, in situ crystallization, or physical mixing—inevitably occludes a fraction of zeolite micropore entrances, reducing the accessible internal surface area and diminishing the contribution of the zeolite component to total adsorption capacity. The severity of this effect scales with carbon loading and is compounded by the tendency of graphene oxide sheets and carbon nanotubes to aggregate on zeolite external surfaces rather than distributing uniformly, producing heterogeneous composites in which local carbon density varies substantially between particles [52,54]. This structural heterogeneity is rarely quantified in published studies, yet it directly affects reproducibility: batch-to-batch variability in carbon distribution translates into variability in adsorption performance that undermines the reliability of reported capacity values.

Regeneration of carbon–zeolite composites present a more complex challenge than regeneration of either component alone. Thermal regeneration temperatures sufficient to combust adsorbed organics from activated carbon domains (400–550 °C) progressively oxidize graphene oxide and biochar phases, reducing their aromatic domain density and hydrophobic character over successive cycles and producing an irreversible decline in π–π stacking and hydrophobic partitioning capacity [56,57]. Solvent-based regeneration preserves composite structure but generates concentrated organic waste streams and is less effective for strongly adsorbed contaminants retained within zeolite micropores, where solvent diffusion is kinetically limited. No regeneration protocol has been demonstrated to achieve consistent capacity recovery above 85% over more than five cycles for any carbon–zeolite composite system treating environmentally relevant OMP concentrations, and the operational lifetime before performance falls below an acceptable threshold remains undefined for all composite configurations reported to date.

Synthesis reproducibility constitutes a further underappreciated limitation, particularly for composites prepared through hydrothermal co-crystallization or chemical vapor deposition routes. The sensitivity of zeolite crystallization kinetics to minor variations in temperature, pH, and precursor concentration means that small deviations in synthesis conditions can produce composites with markedly different zeolite crystallinity, carbon distribution, and pore accessibility—differences that are not always detectable by routine characterization but that substantially affect adsorption performance [49]. The predominance of laboratory-scale synthesis at gram quantities, where precise parameter control is feasible, conceals reproducibility challenges that emerge at larger scales and that have not been systematically evaluated in the current literature.

**Table 2:** Representative carbon–zeolite composite materials investigated for OMP removal from aqueous systems.

Composite Type	Target OMPs	Dominant Adsorption Mechanisms	Key Findings	Reference
Activated carbon–zeolite composite	Carbamazepine, diclofenac	π–π interactions, hydrophobic partitioning	Composite materials showed significantly higher adsorption capacity compared to pristine zeolite due to enhanced surface hydrophobicity; synergistic removal in multicomponent systems	[47]
Carbon-coated zeolite	Sulfamethoxazole	π–π stacking, pore filling	Carbon coating increased adsorption sites; excessive loading reduced pore accessibility of zeolite micropores	[54]
Biochar–zeolite composite	Antibiotics, phenolic compounds	Hydrophobic interactions, hydrogen bonding	Biochar addition improved adsorption efficiency in multicomponent systems; both phases contribute complementary adsorption sites	[58]
Graphene oxide–zeolite composite	Bisphenol A, pharmaceuticals	π–π interactions, electrostatic attraction	GO sheets enhanced adsorption capacity through additional aromatic surface domains anchored to external zeolite surface	[50]
Carbon nanotube–zeolite hybrid	Pharmaceutical residues	π–π stacking, surface adsorption	CNT incorporation improved adsorption kinetics and capacity; high-aspect-ratio aromatic domains reduce intraparticle diffusion path lengths	[40,52]

The data compiled in Table 2 confirm that carbonaceous modification consistently expands the mechanistic repertoire of zeolite-based sorbents beyond ion exchange, with π–π stacking and hydrophobic partitioning emerging as the dominant interaction pathways for aromatic pharmaceutical contaminants across all composite types examined. Notably, the nature of the carbonaceous phase exerts a decisive influence on both the magnitude of adsorption enhancement and the contaminant specificity of the material. Activated carbon composites achieve the broadest applicability through their heterogeneous pore distribution and chemically diverse surface, while graphene oxide composites offer superior performance for aromatic EDCs through their extended two-dimensional π-electron surface. Biochar–zeolite systems present a cost-effectiveness advantage using waste-derived precursors, though their adsorption performance is more variable and strongly dependent on biochar source material and pyrolysis conditions. A critical observation emerging from Table 2 is that performance data across studies are rarely directly comparable: differences in zeolite topology, carbon loading, synthesis method, target contaminant, and experimental conditions—particularly contaminant concentration, pH, and background matrix—preclude quantitative cross-study benchmarking. This lack of standardized evaluation protocols represents a persistent methodological gap in the carbon–zeolite composite literature that substantially complicates the identification of optimal material configurations for specific water treatment applications. Standardized characterization methods developed for carbonaceous adsorbents—including the iodine number (ISO 787-13), molasses number, and methylene blue value—provide operationally defined proxies for micropore volume, mesopore accessibility, and surface area that enable cross-laboratory benchmarking and quality control in industrial practice [46]. No analogous set of consensus test methods has been established for zeolite-based composite materials, where the coexistence of multiple phases with distinct adsorption chemistries renders single-parameter characterization inherently insufficient. Until such standardized protocols are adopted—encompassing minimum requirements for composite characterization (BET surface area, pore size distribution, Si/Al ratio, metal loading), adsorption testing conditions (environmentally representative OMP concentrations, real water matrix, multicomponent systems), and cyclic stability assessment—cross-study comparison will remain limited and the translation of laboratory findings into design criteria for water treatment applications will be impeded.

### 3.3. Polymer- and Surfactant-Modified Zeolite Composites

Polymer and surfactant modification of zeolite frameworks serves a dual purpose that distinguishes it from carbonaceous composite strategies: beyond introducing additional interaction sites for OMP molecules, it simultaneously addresses the practical processability constraints that limit the deployment of powdered zeolites in continuous water treatment systems. The two objectives—enhanced adsorption chemistry and improved engineering applicability—are pursued through distinct modification strategies that merit separate consideration [7].

Surfactant-modified zeolites (SMZ) are prepared by anchoring cationic quaternary ammonium surfactants—most commonly hexadecyltrimethylammonium (HDTMA^+^) or cetylpyridinium chloride (CPC)—onto the negatively charged external zeolite surface through electrostatic adsorption. At surfactant loadings below the external cation exchange capacity of the zeolite, a monolayer of surfactant molecules forms on the surface, presenting outward-facing hydrocarbon tails that create an organophilic surface phase. At loadings exceeding the external CEC, a bilayer configuration develops in which the inner layer of surfactant molecules is electrostatically bound to the zeolite surface while the outer layer is held through hydrophobic tail–tail interactions, with charged head groups facing the aqueous phase [55]. This bilayer architecture is particularly effective for simultaneous removal of hydrophobic organic contaminants—which partition into the hydrocarbon interior of the bilayer—and anionic species such as oxyanions or anionic pharmaceuticals, which interact electrostatically with the positively charged outer surface. SMZ materials have demonstrated effective removal of diclofenac, salicylic acid, and a range of pesticides, with performance governed primarily by the balance between partitioning and electrostatic mechanisms as a function of compound speciation and pH [59,60].

Chitosan-modified zeolite composites represent the most extensively studied biopolymer–zeolite system. The amino (–NH_2_) and hydroxyl (–OH) groups of chitosan introduce pH-responsive surface charge—becoming protonated and positively charged under acidic conditions—enabling electrostatic uptake of anionic contaminants, hydrogen bonding interactions with polar functional groups, and coordination bonding with metal-containing species [59,60]. Chitosan–zeolite composites are typically prepared by immobilising zeolite particles within a cross-linked chitosan matrix through ionic gelation or chemical cross-linking with glutaraldehyde, yielding bead-format materials suitable for column operation. The development of granulated nanosorbent formats based on natural zeolite materials combined with biopolymer matrices has been specifically proposed as a strategy to improve mechanical strength and fixed-bed applicability [60]. Alginate and polyvinyl alcohol (PVA)–based matrices have similarly been employed to encapsulate zeolite particles into mechanically stable composite beads and membranes, improving sorbent recovery and enabling integration into continuous-flow configurations [55].

A critical limitation of the polymer-modified zeolite literature that must be acknowledged is the predominance of studies conducted with model dye contaminants—Congo Red, methylene blue, and similar species—at concentrations of mg L^−1^ to g L^−1^, conditions that bear little resemblance to the ng L^−1^ to µg L^−1^ concentration range of environmentally relevant OMPs [61]. Dye molecules are structurally distinct from pharmaceuticals and EDCs, typically carrying multiple charged groups and large aromatic systems, and their adsorption behaviour cannot be directly extrapolated to trace pharmaceutical removal. This gap between model-system performance and realistic OMP removal represents the most significant unresolved challenge in the polymer–zeolite composite field and substantially limits the practical conclusions that can be drawn from the existing literature. Representative polymer- and surfactant-modified zeolite systems are summarised in Table 3.

The systems summarised in Table 3 illustrate the dual character of polymer and surfactant modification as a composite engineering strategy. Surfactant-modified zeolites achieve their performance through surface chemistry transformation—converting the hydrophilic, negatively charged zeolite surface into an organophilic, positively charged adsorption platform—and are most effective for contaminants whose removal benefits from this reversed surface polarity: hydrophobic pharmaceuticals that partition into the surfactant bilayer, and anionic species that interact electrostatically with the positively charged outer surface. Polymer-matrix composites, by contrast, achieve their primary benefit through improved physical format—mechanical stability, column operability, and sorbent recoverability—rather than through dramatic adsorption chemistry enhancement, and their performance relative to the dispersed zeolite component is often modest in terms of equilibrium capacity.

The reduction of Table 3 to studies reporting performance with pharmaceutically and environmentally relevant OMPs—rather than model dye systems—exposes the limited evidentiary basis currently available for this composite class. Of the three remaining entries, only the diclofenac study on structurally modified NaY [62] reports adsorption performance for a regulated pharmaceutical contaminant; the remaining entries either address broad contaminant categories without specifying OMP-relevant concentration ranges or employ conditions substantially removed from those encountered in real water treatment scenarios. This evidentiary gap has direct consequences for the reliability of existing performance data: dye molecules carry multiple charged groups and large aromatic systems that render their adsorption behaviour mechanistically distinct from trace pharmaceuticals and EDCs at ng L^−1^ to µg L^−1^ concentrations, and removal efficiencies reported for dye-based systems cannot be extrapolated to predict OMP removal performance. Future studies in this composite class should adopt minimum reporting standards including target contaminants drawn from regulated or priority OMP lists, initial concentrations in the ng L^−1^ to low µg L^−1^ range, and evaluation in at least one real or synthetic water matrix containing NOM and background electrolytes at environmentally representative levels [19,57].

### 3.4. Metal-Oxide-Functionalised Zeolite Composites: Coupling Adsorption with Catalytic Degradation

The incorporation of catalytically active metal oxides into zeolite frameworks opens a qualitatively different design dimension relative to carbonaceous and polymeric modifications: rather than simply enhancing adsorption capacity, metal-oxide functionalisation enables the subsequent mineralisation of adsorbed contaminants through photocatalytic or oxidative pathways, addressing the fundamental limitation of purely adsorptive systems—the accumulation of concentrated contaminants on the sorbent surface that eventually necessitates regeneration or disposal.

TiO_2_–zeolite photocatalytic composites constitute the most extensively studied class within this category. Titanium dioxide generates highly reactive hydroxyl radicals (•OH, redox potential +2.80 V vs. NHE) under UV or, in modified forms, visible-light irradiation, capable of non-selectively oxidising a broad range of organic contaminants to CO_2_, H_2_O, and inorganic ions [62,63]. When dispersed on a zeolite support, TiO_2_ nanoparticles achieve more uniform surface distribution and reduced agglomeration compared with unsupported suspensions, increasing the active photocatalytic surface area accessible to incoming photons and contaminant molecules. Crucially, the adsorptive function of the zeolite support creates a spatial concentration effect: contaminant molecules are selectively pre-concentrated within the zeolite pores and on the external surface in the vicinity of TiO_2_ particles, substantially increasing the probability of productive radical–contaminant encounters relative to a homogeneous suspension system [64]. This adsorption–photocatalysis synergy has been documented for diclofenac removal using TiO_2_/zeolite Y composites, for carbamazepine degradation on TiO_2_/ZSM-5, and for pharmaceutical mixtures on TiO_2_/clinoptilolite systems, with removal efficiencies consistently exceeding those of unsupported TiO_2_ under equivalent irradiation conditions [65].

ZnO–zeolite composites operate through a mechanistically analogous photocatalytic pathway but offer the practical advantage of a wider spectral response extending into the near-visible range for certain ZnO morphologies. Reports of ibuprofen and beta-blocker degradation using ZnO/zeolite materials indicate photocatalytic efficiencies of 85–92% under UV irradiation, with the zeolite support improving ZnO stability and inhibiting photocorrosion through structural confinement of ZnO nanoparticles [66,67].

Heterogeneous Fenton and photo-Fenton systems based on iron-functionalised zeolites exploit the Haber–Weiss cycle—the iron-catalysed decomposition of H_2_O_2_ to generate •OH radicals—to achieve oxidative degradation under conditions compatible with water treatment (ambient temperature and pressure, near-neutral pH for heterogeneous systems) [40,68]. Fe-ZSM-5 and Fe-exchanged zeolite Y have demonstrated effective degradation of paracetamol, sulfamethoxazole, and diclofenac through this mechanism, with the zeolite framework stabilising iron species against leaching and providing adsorption sites that concentrate contaminants near catalytically active Fe centres. Magnetic Fe_3_O_4_–zeolite composites combine Fenton-type catalytic activity with the practical benefit of magnetic recoverability: following treatment, the composite can be separated from the treated water using an external magnetic field, eliminating the need for filtration and enabling straightforward reuse [61,69].

Copper and manganese oxide–zeolite composites extend catalytic OMP degradation to non-photocatalytic oxidative pathways. CuO/ZSM-5 and MnO_2_/zeolite systems have been investigated for phenol and dye degradation through heterogeneous catalytic oxidation using H_2_O_2_ or persulfate as oxidants, achieving removal efficiencies of 78–95% depending on oxidant concentration and pH [70,71,72,73]. The zeolite framework again serves a dual function: providing a high-surface-area support for metal oxide dispersion and contributing adsorptive pre-concentration of target molecules.

A recurring challenge across all metal-oxide-functionalised zeolite systems is the trade-off between catalytic metal loading and pore accessibility. Excessive metal oxide deposition partially occludes zeolite micropore entrances, reducing the contribution of the zeolite adsorption mechanism and diminishing the synergistic benefit that motivates the composite approach [74,75]. Optimal metal loading—typically in the range of 5–15 wt% for TiO_2_ and Fe_2_O_3_ systems—must be established empirically for each composite–contaminant combination, and reported optima are not transferable across different zeolite topologies or synthesis methods. Long-term catalytic stability under repeated use cycles, metal leaching under acidic or oxidative conditions, and performance under realistic multicomponent water matrices remain insufficiently characterised in the current literature and represent priority areas for future investigation. Representative metal-oxide–zeolite composite systems and their reported removal efficiencies are summarised in Table 4.

The data compiled in Table 4 reveal a pattern that extends across all three composite classes examined in this section: reported removal efficiencies under laboratory conditions are consistently high, frequently exceeding 85–90%, yet the experimental conditions under which these values are obtained differ fundamentally from those encountered in practice across three distinct dimensions that must be explicitly distinguished.

First, most studies employ single-solute batch systems in deionised or ultrapure water at contaminant concentrations of 5–50 mg L^−1^—conditions that are removed by three to six orders of magnitude from the ng L^−1^ to µg L^−1^ concentrations characteristic of real OMP-contaminated waters. At environmentally relevant trace concentrations, adsorption isotherms operate in the Henry’s law region where capacity expression differs fundamentally from that observed at mg L^−1^ loadings, and competitive adsorption by NOM—present at concentrations several orders of magnitude above individual OMP concentrations—systematically reduces effective uptake in ways that single-solute systems cannot capture [18,19].

Second, batch equilibrium experiments—the dominant experimental format across the studies in Table 4—provide thermodynamic capacity data under conditions of infinite contact time and controlled mixing that are not representative of fixed-bed column operation, where hydraulic residence time, channelling, and mass transfer limitations govern performance. The transition from batch to column operation consistently reduces effective removal relative to equilibrium predictions, and breakthrough behaviour—the operationally critical performance parameter for continuous water treatment—is rarely reported in the zeolite-based composite literature.

Third, real water matrices introduce simultaneous competitive effects from dissolved organic matter, inorganic ions, and co-occurring OMPs that interact in non-additive ways with composite surface chemistry. Studies evaluating zeolite-based composites in secondary wastewater effluent or natural water matrices—rather than laboratory-prepared single-solute systems—consistently report capacity reductions of 30–70% relative to ultrapure water controls, with the magnitude of reduction depending strongly on NOM character, ionic strength, and the dominant adsorption mechanism of the target compound [81]. Until performance evaluation in the field routinely incorporates all three dimensions—environmentally representative concentrations, dynamic flow conditions, and real water matrices—reported removal efficiencies should be interpreted as upper-bound estimates rather than predictors of achievable treatment performance.

## 4. Adsorption Mechanisms Governing OMP Removal by Zeolite-Based Composites

### 4.1. Electrostatic Interactions and Ion Exchange

Electrostatic interactions constitute the primary adsorption mechanism for ionizable OMPs on zeolite frameworks and their functionalized derivatives and arise from the permanent negative charge of the aluminosilicate framework generated by isomorphic Si^4+^ → Al^3+^ substitution. The strength and direction of electrostatic interactions are jointly controlled by the framework charge density—expressed through the Al content and Si/Al ratio—and the charge state of the contaminant molecule at the prevailing solution pH, which is in turn determined by the compound’s pKa values as established in Section 2.2.

Cationic pharmaceuticals—including protonated β-blockers such as atenolol and metoprolol (pKa ~9.6), and aminoglycoside antibiotics with multiple protonated amine groups—interact favorably with the negatively charged zeolite surface through direct electrostatic attraction and ion exchange, displacing pre-existing extra-framework cations from exchange positions. This mechanism is thermodynamically favorable at circumneutral pH where these compounds carry net positive charge, and adsorption capacities in this regime can be substantial: ion-exchange-driven uptake of cationic pharmaceuticals on clinoptilolite and zeolite Y has been reported at maximum capacities of 20–80 mg g^−1^ under optimized conditions [40,68]. However, the same electrostatic affinity renders these systems highly sensitive to competition from dissolved inorganic cations—Na^+^, Ca^2+^, Mg^2+^—which are present at concentrations several orders of magnitude higher than pharmaceutical contaminants in natural and wastewater matrices, and effectively suppress ion-exchange-driven pharmaceutical uptake under realistic conditions.

For anionic pharmaceuticals—diclofenac, ibuprofen, sulfamethoxazole, and PFAS at pH 7—the negatively charged zeolite framework presents an electrostatic barrier rather than an attraction. Adsorption of these species on unmodified zeolites is therefore limited and occurs primarily through non-electrostatic mechanisms. Metal-oxide functionalization—introducing positively charged TiO_2_, Fe_2_O_3_, or Al_2_O_3_ surface sites—and surfactant bilayer modification—presenting a cationic outer surface—both reverse this electrostatic relationship, enabling effective uptake of anionic OMPs through surface complexation or electrostatic attraction to the modified sorbent surface [50]. The pH-dependence of this interaction is pronounced: as solution pH increases above the point of zero charge of the metal oxide surface (pH_pzc ~5.8 for TiO_2_, ~8.1 for Fe_2_O_3_), surface sites progressively deprotonate and become less effective for anionic OMP capture, producing the characteristic decrease in removal efficiency with increasing pH that is consistently reported for metal-oxide-modified zeolite systems [46].

The quantitative relationship between Si/Al ratio and adsorption performance is compound-class-specific and cannot be reduced to a single directional trend. For neutral, hydrophobic OMPs—including carbamazepine (log K_ow_ 2.45) and bisphenol A (log K_ow_ 3.32)—increasing Si/Al ratio systematically enhances adsorption capacity by reducing framework polarity and competitive hydration within micropore channels. Jiang et al. reported that high-silica zeolites (Si/Al > 200) achieved carbamazepine uptake approximately 3–5 times higher than low-silica clinoptilolite (Si/Al ~5) under equivalent conditions, attributing this to the near-elimination of strongly bound water molecules from the hydrophobic micropore interior [15]. For ionizable pharmaceuticals whose removal depends substantially on electrostatic interactions—including cationic metformin and anionic sulfamethoxazole—this relationship inverts: reducing the Si/Al ratio increases framework charge density and ion-exchange capacity, enhancing uptake of cationic species while the reduced hydrophobicity simultaneously diminishes performance toward neutral contaminants. Quantitative cross-composite comparison further reveals that the Si/Al effect is modulated by the nature of the secondary phase: in carbon–zeolite composites, the carbonaceous phase compensates for reduced zeolite hydrophobicity at low Si/Al ratios through its own hydrophobic partitioning capacity, effectively decoupling composite performance from the Si/Al ratio for hydrophobic OMPs [48,52]. In metal-oxide-functionalized composites, the TiO_2_ or Fe_2_O_3_ surface chemistry dominates electrostatic interactions to an extent that renders the zeolite Si/Al ratio a secondary rather than primary determinant of anionic OMP removal efficiency [83]. These quantitative trends, summarized across composite classes and OMP types in Table 1, underscore that Si/Al ratio optimization must be conducted in conjunction with—not independently of—the selection of the secondary functional phase and the molecular properties of the target contaminant class.

### 4.2. Hydrophobic Interactions and Partitioning

For neutral, non-ionisable organic contaminants—and for ionisable compounds under pH conditions where their net charge approaches zero—hydrophobic interactions become the dominant adsorption driving force. The thermodynamic basis of hydrophobic adsorption is the enthalpically and entropically favourable transfer of a nonpolar molecular surface from the aqueous phase, where it disrupts hydrogen-bond networks among water molecules, into a nonpolar sorbent domain where such disruption does not occur. The magnitude of this driving force scales with the accessible nonpolar molecular surface area of the contaminant and the hydrophobicity of the sorbent domain, making log Kow the primary molecular predictor of hydrophobic adsorption affinity [29,30].

In carbon-modified zeolite composites, the carbonaceous phase provides the primary hydrophobic adsorption domain: the basal plane surfaces of graphite-like structures in activated carbon and biochar, and the outer wall surfaces of carbon nanotubes, present extended nonpolar regions with which hydrophobic contaminant molecules interact through van der Waals forces and hydrophobic partitioning. High-silica zeolite frameworks contribute a secondary hydrophobic environment within their micropore channels, where the reduced density of silanol groups and absence of framework aluminium create an internal surface of low polarity. The synergistic contribution of both phases to neutral OMP removal has been demonstrated for carbamazepine (log K_ow_ = 2.45) and bisphenol A (log K_ow_ = 3.32) on activated carbon–zeolite composites, where measured adsorption capacities exceeded additive predictions based on single-component performance [60].

For PFAS, hydrophobic partitioning of the perfluorinated chain into nonpolar sorbent domains is a primary but not sole adsorption mechanism. The strong electron-withdrawing effect of fluorine substituents renders the C–F bonds highly polarised and the perfluorinated chain less hydrophobic per carbon atom than the equivalent hydrocarbon chain, which explains why PFAS adsorption on carbonaceous materials does not scale linearly with chain length in the manner predicted by simple hydrophobicity models [31]. Effective PFAS removal by composite zeolites therefore requires materials that engage both the perfluorinated chain through hydrophobic interactions and the charged head group through electrostatic attraction—a dual-mode requirement that constrains composite design and has motivated the development of purpose-engineered materials incorporating both hydrophobic and positively charged surface domains.

### 4.3. π–π Stacking Interactions

π–π stacking between aromatic ring systems of OMP molecules and graphitic aromatic domains of carbonaceous composite phases constitutes a mechanistically distinct and quantitatively significant adsorption pathway for the large proportion of pharmaceuticals and EDCs that contain aromatic or heteroaromatic ring systems. These interactions arise from quadrupolar electrostatic forces between the π-electron clouds of face-to-face or offset-stacked aromatic rings, with interaction energies of approximately 2–8 kJ mol^−1^ per ring pair for typical pharmaceutical–graphene systems [37,38]. While individually modest, π–π interactions operate additively across multiple ring pairs and in concert with hydrophobic and van der Waals forces, producing a combined interaction energy that substantially enhances adsorption affinity for polycyclic aromatic contaminants.

The relative contributions of π–π stacking and hydrophobic partitioning to the total adsorption free energy are difficult to deconvolute experimentally, and the two mechanisms frequently co-operate for the same compound. Carbamazepine, with its dibenzazepine ring system, interacts with graphitic surfaces through both mechanisms simultaneously, and its anomalously strong adsorption on carbon-modified zeolites relative to its moderate log Kow value has been attributed to this dual contribution [56,60]. Electron-withdrawing substituents on the contaminant aromatic ring—as in fluoroquinolone antibiotics—can modulate π–π stacking geometry by shifting the electron density distribution of the aromatic system, creating donor–acceptor π–π interactions with electron-rich graphitic domains that differ in geometry and energy from the symmetric stacking of unsubstituted aromatics [38].

### 4.4. Hydrogen Bonding

Hydrogen bonding between OMP functional groups and surface hydroxyl, amine, or carbonyl groups of polymer-modified zeolite composites provides an adsorption pathway that is particularly relevant for polar pharmaceuticals carrying amide, sulfonamide, hydroxyl, or carboxyl moieties. Although individual hydrogen bonds (interaction energy 10–40 kJ mol^−1^) are stronger than individual π–π or hydrophobic interactions, their geometric specificity—requiring precise donor–acceptor distance and angle constraints—means that not all polar functional groups engage in hydrogen bonding with a given sorbent surface, and that the contribution of hydrogen bonding to total adsorption affinity is highly compound- and surface-specific [39].

Chitosan-modified zeolite composites present a particularly rich hydrogen-bonding environment: the –NH_2_ and –OH groups of chitosan can act simultaneously as hydrogen-bond donors and acceptors, enabling interaction with both electron-rich and electron-deficient functional groups of OMP molecules. Sulfamethoxazole adsorption on chitosan–zeolite composites has been attributed in part to hydrogen bonding between the sulfonamide –NH– group of the antibiotic and the –OH groups of the chitosan matrix, supplementing electrostatic interactions operative under specific pH conditions [66]. Similarly, the silanol groups (Si–OH) present on the external surface of zeolite frameworks—particularly in high-silica variants where framework charge is low—provide hydrogen-bonding sites for contaminants carrying electron-donating functional groups, contributing to the adsorption of compounds such as atrazine and certain phenolic EDCs [40].

### 4.5. Pore Confinement and Molecular Sieving

The microporous architecture of zeolite frameworks introduces a mechanistically unique adsorption contribution—pore confinement—that has no direct analogue in amorphous carbonaceous or polymeric sorbents. When the kinetic diameter of an OMP molecule closely matches the pore aperture dimensions of the zeolite framework, adsorption within the micropore channel is accompanied by a confinement energy contribution arising from the maximisation of van der Waals contact between the molecule and the surrounding channel walls [45]. This confinement effect can substantially enhance adsorption affinity for size-compatible molecules, producing steep adsorption isotherms and high selectivity for specific contaminant geometries. Conversely, molecules whose kinetic diameter exceeds the pore aperture are excluded from the micropore volume entirely, limiting adsorption to the external surface and dramatically reducing effective capacity.

The practical implications of molecular sieving for OMP removal are twofold. On one hand, size-selective exclusion of larger competing molecules—including humic substance fragments and large natural organic matter components—from zeolite micropores can provide a degree of protection against competitive adsorption that is not available to amorphous sorbents with broad pore size distributions. On the other hand, the same size selectivity restricts access to the micropore volume for larger pharmaceutical molecules, including macrolide antibiotics and steroid hormones with effective diameters approaching or exceeding 1 nm, confining their adsorption to the external surface area of the zeolite particles [35,45]. The development of hierarchical zeolite composites incorporating additional mesoporosity—through controlled desilication, templating, or assembly of zeolite nanocrystals—addresses this limitation by providing interconnected transport pathways that improve molecular diffusion toward micropore adsorption sites while preserving the size-selective confinement benefit of the microporous framework.

### 4.6. Mechanistic Interplay and Implications for Composite Design

**Figure 3:**
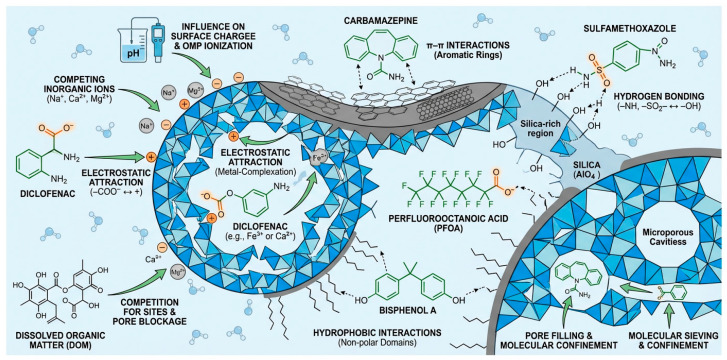
Schematic illustration of the five dominant adsorption mechanisms governing OMP removal by zeolite-based composite materials: electrostatic attraction, π–π stacking, hydrogen bonding, hydrophobic partitioning, and pore confinement within zeolite micropore channels. The relative contribution of each mechanism depends on OMP molecular structure, composite surface chemistry, and solution pH.

The five interaction mechanisms described in Section 4.1, Section 4.2, Section 4.3, Section 4.4 and Section 4.5 do not operate independently but simultaneously and interdependently, with their relative contributions shifting as a function of contaminant molecular structure, composite surface chemistry, solution pH, and water matrix composition. A schematic representation of the dominant adsorption mechanisms and their relationship to composite architecture is presented in Figure 3.

As illustrated in Figure 3, the five interaction mechanisms operate neither independently nor with fixed relative contributions: their hierarchy shifts continuously as a function of contaminant speciation, sorbent surface chemistry, and water matrix composition. For a given composite–contaminant pair, the dominant mechanism at pH 5 may differ fundamentally from that at pH 8, and performance measured in deionized water may reflect an entirely different mechanistic balance than that operating in secondary wastewater effluent. This pH- and matrix-dependence of mechanistic hierarchy has a direct consequence for the interpretation of adsorption data: single-point isotherm measurements or removal efficiency values obtained under a fixed set of laboratory conditions provide limited predictive power for performance under the variable conditions encountered in real water treatment systems. A further implication of the mechanistic framework developed in this section concerns the design logic of multi-component composite architectures. Ternary composites combining a carbonaceous phase, a zeolite framework, and a metal oxide component—such as TiO_2_–biochar–zeolite or Fe_3_O_4_–GO–zeolite systems—engage OMP molecules through four or five simultaneous interaction pathways: hydrophobic partitioning and π–π stacking through the carbonaceous phase, electrostatic and ion-exchange interactions through the zeolite framework and metal oxide surface sites, hydrogen bonding through surface hydroxyl and functional groups, and photocatalytic or Fenton-type mineralization through the metal oxide catalytic centers. This mechanistic breadth confers two practical advantages: broader contaminant scope encompassing both hydrophobic neutral and ionizable charged OMPs, and greater resilience to water matrix variability, since the suppression of one interaction pathway by a specific matrix component—for example, electrostatic shielding by high ionic strength—is compensated by the continued contribution of matrix-insensitive pathways such as π–π stacking and hydrophobic partitioning. The translation of this mechanistic understanding into quantifiable performance outcomes under realistic water matrix conditions is examined in the following section.

### 4.7. PFAS Adsorption: A Mechanistically Distinct Case

Per- and polyfluoroalkyl substances occupy a mechanistically unique position among OMP classes addressed in this review, and their removal by zeolite-based composites cannot be adequately described within the mechanistic framework developed for pharmaceuticals and EDCs in Section 4.1, Section 4.2, Section 4.3, Section 4.4, Section 4.5 and Section 4.6. Three structural features collectively define this distinctiveness and impose specific constraints on composite design.

First, the exceptional strength of the carbon–fluorine bond (bond dissociation energy ~544 kJ mol^−1^) renders PFAS essentially inert to chemical and biological degradation under environmental conditions [22], eliminating oxidative degradation as a viable treatment pathway under mild conditions and placing the entire removal burden on adsorptive mechanisms. Second, the amphiphilic molecular architecture of long-chain PFAS—a perfluorinated hydrophobic tail paired with a charged hydrophilic head group (carboxylate in PFOA, sulfonate in PFOS)—produces a dual-mode adsorption requirement that is not met by any single interaction pathway: effective removal requires simultaneous engagement of the perfluorinated chain through hydrophobic interactions and the charged head group through electrostatic attraction [30,36]. Third, the strong electron-withdrawing effect of fluorine substituents renders the C–F bonds highly polarized, making the perfluorinated chain less hydrophobic per carbon atom than the equivalent hydrocarbon chain; as a consequence, PFAS adsorption on carbonaceous materials does not scale linearly with chain length in the manner predicted by conventional hydrophobicity models based on log K_ow_, and short-chain PFAS (C4–C6) are substantially more difficult to remove than their long-chain analogues [31].

These structural constraints translate directly into composite design requirements. Carbon–zeolite composites provide hydrophobic domains for perfluorinated chain interaction, but their performance for PFAS is consistently inferior to that observed for aromatic pharmaceuticals of comparable log K_ow_, reflecting the reduced hydrophobicity per carbon unit of fluorinated chains relative to hydrocarbon analogues [43]. Effective PFAS capture therefore requires materials incorporating both hydrophobic and positively charged surface domains simultaneously—a combination most readily achieved through surfactant-modified zeolites presenting a cationic bilayer surface, or through amine-functionalized composite architectures that engage the anionic PFAS head group electrostatically while hydrophobic domains interact with the fluorinated tail [36,44]. The molecular sieving properties of small-pore zeolite frameworks have additionally been proposed as a mechanism for selective PFAS capture based on chain-length discrimination, with framework apertures of 0.5–0.6 nm offering preferential access to short-chain PFAS while excluding competing NOM macromolecules [36].

Water matrix effects on PFAS adsorption differ qualitatively from those observed for other OMP classes. NOM competition—the dominant capacity-reducing factor for pharmaceutical adsorption on carbon–zeolite composites—exerts a comparatively modest influence on PFAS uptake by electrostatic-hydrophobic dual-mode materials, because the combination of two simultaneous interaction pathways provides greater resilience to competitive displacement than single-mechanism adsorption [30]. Ionic strength effects, by contrast, are particularly pronounced for PFAS: increasing concentrations of monovalent and divalent background cations compete directly with the positively charged composite surface for interaction with the anionic PFAS head group, reducing electrostatic uptake efficiency under the high-ionic-strength conditions characteristic of contaminated groundwater and industrial effluents [44]. Regulatory limits in the sub-ng L^−1^ range now applicable in several jurisdictions [23] impose extreme demands on adsorption capacity and place PFAS removal among the most technically demanding applications for zeolite-based composite materials, warranting dedicated experimental investigation under conditions specifically representative of PFAS-contaminated source waters.

## 5. Influence of Water Matrix and Operational Conditions on OMP Adsorption

### 5.1. Solution pH

Solution pH is the single most influential operational parameter governing OMP adsorption on zeolite-based composite materials, because it simultaneously controls two interdependent variables: the surface charge of the adsorbent and the speciation—and therefore the net charge—of ionizable contaminant molecules. The combined pH-dependence of both variables produces adsorption–pH relationships that are non-linear, compound-specific, and frequently non-monotonic, requiring explicit mechanistic interpretation rather than simple empirical correlation.

For metal-oxide-functionalized zeolite composites, the pH-dependence of surface charge is governed by the point of zero charge (pH_pzc) of the metal oxide phase. At solution pH below pH_pzc, metal oxide surface sites are protonated and carry net positive charge, favoring electrostatic adsorption of anionic OMPs; above pH_pzc, surface deprotonation generates net negative charge, suppressing anionic OMP uptake and, in some cases, enhancing adsorption of cationic species. For TiO_2_-modified zeolites (pH_pzc ~5.8) and Fe_2_O_3_-functionalized systems (pH_pzc ~8.1), the pH window of effective anionic pharmaceutical removal differs substantially, with iron-oxide composites maintaining favorable surface charge for anionic diclofenac and ibuprofen removal across a broader range of environmentally relevant pH values [84]. For the unmodified aluminosilicate framework, whose surface carries permanent negative charge independent of pH, electrostatic interactions consistently favor cationic OMP adsorption and disfavor anionic species across the full pH range of natural waters.

Contaminant speciation introduces an additional layer of pH-dependence that is compound-specific and directly linked to the pKa values established in Section 2.2. Diclofenac (pKa 4.0) transitions from its neutral acid form to the anionic diclofenac anion at pH values above approximately 5, reducing its hydrophobicity and altering the balance between hydrophobic and electrostatic adsorption mechanisms. Sulfamethoxazole, with two pKa values (1.6 and 5.7), exists as a cation below pH 1.6, as a neutral zwitterion between pH 1.6 and 5.7, and as an anion above pH 5.7—producing a characteristic adsorption maximum near its neutral point that is consistently observed across multiple sorbent types [32,33]. Fluoroquinolone antibiotics present the most complex pH-adsorption relationship of any pharmaceutical class due to their amphoteric character, with adsorption maxima typically observed near the pH of net zero charge of the molecule (~pH 6–7 for ciprofloxacin) where hydrophobic and π–π stacking mechanisms operate without electrostatic repulsion [34].

### 5.2. Natural Organic Matter

The presence of natural organic matter in source and treated waters represents the most universally documented cause of reduced OMP adsorption capacity in zeolite-based composite systems under realistic conditions, and its effects operate through three mechanistically distinct pathways that act simultaneously and are difficult to deconvolute experimentally.

Competitive adsorption at the same surface sites arises when NOM components—particularly hydrophobic humic acid fractions with log K_ow_ values comparable to those of target OMPs—compete directly for hydrophobic adsorption domains on carbonaceous composite phases. Given that NOM is typically present at dissolved organic carbon concentrations of 2–15 mg L^−1^ in natural waters, several orders of magnitude above individual OMP concentrations, competitive displacement of trace pharmaceuticals from hydrophobic surface sites is thermodynamically highly favorable [78]. Reported reductions in carbamazepine and diclofenac adsorption capacity in the presence of humic acid at environmentally representative concentrations range from 30 to 70% relative to ultrapure-water conditions, depending on composite type and NOM character [85].

Pore blocking occurs when large NOM macromolecules—humic aggregates with hydrodynamic diameters of 1–10 nm—deposit at zeolite micropore entrances, physically obstructing access of OMP molecules to internal adsorption sites. This mechanism is particularly damaging for microporous zeolite composites whose adsorption capacity is concentrated within the micropore volume and is largely irreversible under typical operating conditions since NOM desorption requires conditions incompatible with water treatment [42,43]. Hierarchical zeolite composites with additional microporosity are less susceptible to this pathway because blocked micropore entrances can be partially compensated by adsorption at mesopore walls and external surface sites.

Surface chemistry modification through NOM adsorption can alter both the charge and hydrophobicity of composite surfaces, indirectly affecting OMP–sorbent interactions. NOM adsorption onto positively charged metal-oxide-modified zeolite surfaces reduces the net positive surface charge and thus the electrostatic driving force for anionic OMP uptake—a particularly important effect for TiO_2_ and Fe-zeolite composites designed specifically for anionic pharmaceutical removal [79]. Conversely, NOM adsorption onto hydrophobic high-silica zeolite surfaces can paradoxically enhance uptake of certain hydrophobic OMPs by increasing the apparent hydrophobic surface area available for partitioning, though this effect is contaminant-specific and not consistently observed [44].

### 5.3. Ionic Strength and Competing Inorganic Ions

The influence of ionic strength on OMP adsorption by zeolite-based composites is mechanistically ambiguous and does not produce a universally directional effect, because it operates through competing pathways whose relative importance depends on the dominant adsorption mechanism operative for the specific contaminant–composite combination.

For electrostatically driven adsorption—the dominant mechanism for cationic pharmaceuticals on unmodified zeolites and for anionic OMPs on metal-oxide-functionalized composites—increasing ionic strength compresses the electrical double layer surrounding the charged sorbent surface, reducing the range and magnitude of electrostatic interactions between the surface and approaching contaminant ions. High concentrations of monovalent background electrolytes (NaCl, NaHCO_3_) thus typically suppress electrostatic OMP uptake, with reported capacity reductions of 15–40% at ionic strengths representative of hard drinking water (10–50 mM) [38,39]. The effect is more pronounced for divalent background electrolytes due to their greater charge-shielding efficiency.

Multivalent cations—particularly Ca^2+^ and Mg^2+^ at the concentrations encountered in moderately hard to hard natural waters (1–5 mM)—introduce the additional possibility of cation bridging: coordination of the divalent cation between a negatively charged sorbent surface site and a negatively charged functional group of an anionic OMP molecule, effectively converting an electrostatically repulsive interaction into an attractive one [38]. This bridging mechanism has been invoked to explain observations of enhanced diclofenac and ibuprofen adsorption on zeolite-based composites in the presence of Ca^2+^ relative to Na^+^ at equivalent ionic strength and represents a case where increasing water hardness improves rather than diminishes adsorption performance. For hydrophobically driven adsorption—dominant for neutral, nonpolar OMPs on carbonaceous composite phases—ionic strength effects are generally minor, since the hydrophobic driving force is largely independent of electrostatic screening.

Direct ion competition for zeolite exchange sites between dissolved inorganic cations and cationic pharmaceutical contaminants is quantitatively significant given the concentration disparity: Na^+^, Ca^2+^, and Mg^2+^ typically occur at concentrations of 0.1–10 mM in natural waters, compared with pharmaceutical concentrations of 1–1000 ng L^−1^. Even at modest selectivity coefficients, the thermodynamic preference of zeolite exchange sites for inorganic cations at such extreme concentration ratios effectively suppresses ion-exchange-driven pharmaceutical uptake under realistic conditions, and this mechanism should be considered largely inoperative for cationic OMP removal from natural water matrices [45,46].

### 5.4. Operational Parameters: Contact Time, Temperature, and Sorbent Dosage

Adsorption kinetics in zeolite-based composite systems are governed by a sequence of mass transfer resistances: external film diffusion across the hydrodynamic boundary layer surrounding the sorbent particle, intraparticle diffusion through the macropore and mesopore network toward micropore adsorption sites, and surface adsorption at the final binding site. For microporous zeolite composites, intraparticle diffusion through the micropore channels frequently constitutes the rate-limiting step, producing the characteristic slow approach to equilibrium—often requiring 2–24 h to reach 90% of equilibrium capacity—that limits the practical applicability of microporous adsorbents in high-throughput continuous-flow systems [84]. Hierarchical composite architectures that introduce microporosity reduce intraparticle diffusion path lengths and substantially accelerate kinetics, with equilibrium times of 30–60 min reported for hierarchical zeolite composites compared with several hours for their purely microporous counterparts.

Temperature effects on OMP adsorption are thermodynamically governed and contaminant specific. For exothermic adsorption processes—predominant for hydrophobic OMP partitioning and electrostatic interactions—increasing temperature reduces equilibrium capacity by shifting the thermodynamic balance toward desorption, while simultaneously improving mass transfer kinetics. For endothermic adsorption, where contaminant uptake is entropy-driven—as has been reported for certain pharmaceutical adsorption processes on high-silica zeolites where micropore confinement provides significant entropic gain—increasing temperature enhances both kinetics and equilibrium capacity [41]. The temperature range of practical relevance for water treatment (5–30 °C) typically produces modest capacity changes of 10–25% relative to standard laboratory conditions (20–25 °C), which is significant but secondary to the effects of pH and NOM discussed above.

The influence of water matrix parameters on OMP adsorption across composite types is summarized in Table 5.

As shown in Table 5, adsorption behavior of organic micropollutants may vary significantly depending on water matrix conditions. Among these parameters, solution pH is often considered the most critical factor because it simultaneously influences the ionization state of the contaminant molecules and the surface charge of the adsorbent. Natural organic matter typically reduces adsorption efficiency due to competition for adsorption sites and pore blockage, whereas the influence of ionic strength depends on the balance between electrostatic interactions and ion bridging effects. These observations highlight the importance of evaluating adsorption performance under realistic water conditions when assessing the applicability of zeolite-based composite materials for water treatment.

## 6. Regeneration, Long-Term Stability, and Pathways to Practical Implementation

### 6.1. Regeneration Strategies and Their Limitations

The practical viability of any adsorption-based water treatment technology is determined not by single-cycle removal efficiency but by the capacity retention and operational stability achievable over repeated adsorption–desorption cycles. For zeolite-based composite adsorbents, regeneration presents a fundamentally more complex challenge than for single-component materials such as granular activated carbon, because the composite architecture introduces multiple phases with different thermal stabilities, chemical susceptibilities, and regeneration requirements that may be mutually incompatible.

Thermal regeneration—calcination at 300–550 °C to combust adsorbed organic contaminants—is the most effective method for restoring adsorption capacity in purely inorganic zeolite frameworks and TiO_2_-functionalized composites, which retain structural integrity at elevated temperatures. Reported capacity recoveries of 85–95% per cycle has been documented for high-silica ZSM-5 and zeolite Y systems regenerated by calcination at 400–500 °C [89]. However, this approach is incompatible with polymer-modified and biochar–zeolite composites, where the organic functional phases that provide adsorption enhancement are irreversibly destroyed at regeneration temperatures, eliminating the composite’s competitive advantage over the parent zeolite after the first thermal cycle. Carbon-modified zeolite composites occupy an intermediate position: graphene oxide and activated carbon phases survive moderate thermal treatment (250–350 °C) with partial capacity recovery, but progressive oxidation of the carbonaceous phase over multiple cycles gradually reduces its contribution to total adsorption capacity.

Solvent regeneration—elution of adsorbed OMPs using methanol, ethanol, acetonitrile, or dilute acid/base solutions—preserves the composite structure and is applicable across all composite classes. Reported regeneration efficiencies for solvent-based protocols range widely: methanol elution achieves 70–90% recovery for hydrophobically adsorbed neutral pharmaceuticals on carbon–zeolite composites, while dilute NaOH (0.1 M) effectively desorbs anionic pharmaceuticals from metal-oxide-modified surfaces by converting them to their more hydrophilic deprotonated forms. A critical limitation of solvent regeneration is the generation of a concentrated organic waste stream requiring further treatment or disposal, which introduces a secondary environmental burden and an associated cost that is rarely accounted for in laboratory feasibility assessments. Furthermore, repeated exposure to organic solvents can gradually extract surfactant bilayers from SMZ composites and leach polymer functional groups from chitosan-modified systems, progressively degrading composite surface chemistry over multiple regeneration cycles.

Photocatalytic in situ regeneration—the most conceptually attractive approach for metal-oxide-functionalized zeolite composites—exploits the catalytic activity of TiO_2_ or Fe-oxide phases to mineralize adsorbed contaminants directly on the sorbent surface under UV or solar irradiation, restoring adsorption sites without the need for desorption or external eluents. When the adsorption–photocatalysis coupling is effective, the composite undergoes continuous self-regeneration during irradiation phases, enabling extended operational lifetimes without discrete regeneration cycles. Experimental demonstrations of this concept for TiO_2_/zeolite composites treating diclofenac and carbamazepine have reported capacity retention of 80–92% over five consecutive adsorption–irradiation cycles. However, the rate of photocatalytic mineralization must match or exceed the rate of contaminant adsorption to prevent progressive surface saturation—a balance that is rarely achieved under realistic water matrix conditions where NOM competes for photogenerated radicals and attenuates UV penetration.

Advanced oxidation regeneration using H_2_O_2_ combined with UV irradiation or ozone can restore adsorption capacity of carbon–zeolite composites through oxidative mineralization of surface-bound organic contaminants without thermal treatment. Capacity recoveries of 75–88% over three to five cycles have been reported for AC–zeolite composites regenerated with H_2_O_2_/UV, though partial oxidation of the carbonaceous phase itself—reducing its surface area and hydrophobicity—represents a gradual and irreversible capacity loss mechanism that limits the number of effective regeneration cycles.

### 6.2. Structural Stability and Leaching Concerns

Long-term structural integrity under repeated use cycles and exposure to variable water chemistry conditions is a prerequisite for practical deployment that is substantially under characterized in the current zeolite-based composite literature. Three distinct degradation pathways warrant explicit consideration.

Framework dealumination—the hydrolytic removal of aluminum from zeolite tetrahedral positions under acidic conditions or hydrothermal stress—reduces framework charge density, modifies pore dimensions, and generates extra framework aluminum species that can partially occlude micropore channels. For composites intended for use in slightly acidic water matrices (pH 4–6), or subjected to acid-based regeneration protocols, dealumination represents a realistic long-term stability concern, particularly for low-silica zeolites such as clinoptilolite and zeolite A. High-silica frameworks (Si/Al > 20) exhibit substantially greater hydrolytic stability and are more appropriate for applications involving variable or acidic pH conditions [90].

Metal leaching from metal-oxide-functionalized zeolite composites—the release of Fe, Ti, Cu, or Mn species into treated water—presents both a performance and an environmental safety concern. Iron leaching from Fe-zeolite Fenton catalysts under acidic regeneration conditions (pH < 4) has been reported at concentrations of 0.5–5 mg L^−1^, potentially exceeding drinking water limits and introducing secondary contamination. Titanium leaching from TiO_2_/zeolite composites is generally lower due to the chemical inertness of TiO_2_ but is not negligible under strongly acidic or alkaline conditions. Rigorous leaching characterization under both operating and regeneration conditions—currently absent from most published studies—is an essential prerequisite for regulatory acceptance of metal-oxide-composite technologies in drinking water treatment applications.

Carbonaceous phase degradation through oxidative erosion during photocatalytic or advanced oxidation regeneration cycles progressively reduces the surface area and aromatic domain density of carbon-modified composites, diminishing their capacity for π–π stacking and hydrophobic adsorption. Quantitative assessment of carbonaceous phase integrity—through BET surface area measurement, Raman spectroscopy, and elemental analysis—over extended cycling has been reported in only a limited number of studies, and the operational lifetime before performance degradation becomes unacceptable remains undefined for most composite configurations [56,57].

### 6.3. Scalability, Process Integration, and Techno-Economic Considerations

The transition from laboratory-scale composite synthesis to technically feasible, economically competitive water treatment processes require addressing a series of engineering challenges that are systematically absent from the peer-reviewed literature, where batch experiments at gram-scale with model water matrices dominate.

Most high-performance zeolite-based composites described in the literature are synthesized through multi-step procedures—hydrothermal zeolite crystallization, carbonaceous phase preparation or modification, surface functionalization, and optional granulation or shaping—that are difficult to reproduce at scale without loss of the structural and surface chemistry characteristics that underpin laboratory performance. The use of laboratory-grade chemical precursors, precise temperature and pH control during synthesis, and extended synthesis times of 24–72 h collectively render direct scale-up economically prohibitive for water treatment applications where sorbent cost must compete with granular activated carbon at €1–3 per kg [46,84]. Future synthesis strategies must therefore priorities low-cost, earth-abundant precursors—natural zeolites, industrial zeolite by-products, agricultural biochar—and simplified, reproducible preparation protocols compatible with industrial manufacturing conditions.

Fixed-bed column operation—the configuration of practical relevance for continuous water treatment—introduces hydraulic performance requirements that batch-synthesized powdered composites typically cannot meet adequate mechanical strength to withstand compaction and hydraulic pressure, particle size distributions that balance pressure drop against mass transfer efficiency, and resistance to attrition under sustained flow conditions. Granulation and palletization of composite materials into millimeter-scale particles using binders compatible with the composite surface chemistry—a challenge that is non-trivial for metal-oxide-functionalized and polymer-modified composites—represents a necessary but largely unaddressed engineering development step [65,72].

Techno-economic analysis and life-cycle assessment of zeolite-based composite adsorbents for OMP removal at full scale are conspicuously absent from the current literature. The factors governing economic competitiveness—synthesis cost, operational lifetime, regeneration frequency and cost, waste stream management, energy consumption, and OMP removal performance under realistic conditions—have not been systematically evaluated for any composite type, precluding meaningful comparison with established technologies such as ozonation combined with biological activated carbon filtration, which represents the current state-of-the-art for OMP removal in advanced water treatment [9,10]. Until such analyses are available, claims regarding the practical and economic viability of zeolite-based composite adsorbents remain speculative, and the field would benefit substantially from collaborative studies between materials scientists and water treatment engineers that bridge the gap between proof-of-concept synthesis and application-oriented performance evaluation.

## 7. Research Gaps and Future Directions

The analysis presented in Section 3, Section 4, Section 5 and Section 6 identifies five priority research directions whose systematic pursuit is necessary to advance zeolite-based composite adsorbents from proof-of-concept materials toward deployable water treatment technologies.

**Standardized evaluation under realistic conditions**. The most consequential methodological gap in the current literature is the near-universal use of single-solute, ultrapure-water systems for adsorption performance assessment. The field requires adoption of standardized evaluation protocols incorporating environmentally representative OMP concentrations (ng L^−1^ to low µg L^−1^), real or synthetic water matrices containing NOM at 5–10 mg L^−1^ DOC, competing ions at realistic ionic strength, and multicomponent contaminant mixtures representative of municipal wastewater effluent composition. Without such standardization, cross-study comparison remains impossible, and the body of published adsorption capacity data is of limited practical utility.

**Mechanistic quantification of multi-pathway adsorption**. While the co-existence of multiple adsorption mechanisms in zeolite-based composites is widely acknowledged, their individual quantitative contributions to total adsorption affinity are rarely deconvoluted. Development of experimental protocols that selectively suppress individual interaction pathways—controlled pH variation to isolate electrostatic contributions, competitive displacement experiments to quantify hydrophobic partitioning, molecular modelling to estimate π–π interaction energies—would substantially improve mechanistic understanding and provide rational guidance for composite design optimization.

**Rigorous cyclic stability and leaching assessment**. Long-term performance characterization over a minimum of 10–20 adsorption–regeneration cycles, combined with systematic metal leaching quantification under both operating and regeneration conditions, should be established as a minimum reporting standard for composite materials proposed for water treatment applications. Studies reporting single-cycle or three-cycle reusability data—as is currently the norm—provide insufficient basis for assessing practical deployment potential.

**Rational design of hierarchical multifunctional composites**. The mechanistic analysis developed in this review indicates that composite architectures engaging multiple simultaneous interaction pathways—combining hydrophobic carbonaceous domains, electrostatic metal-oxide surface sites, and catalytic degradation capability—offer the broadest contaminant scope and greatest resilience to water matrix variability. Future materials development should prioritize hierarchical ternary composites in which microporous zeolite frameworks are integrated with mesoporous carbonaceous phases and catalytically active metal oxide nanoparticles, with explicit optimization of interfacial contact quality, pore accessibility, and the balance between adsorptive and catalytic functions. The use of natural zeolites and waste-derived carbonaceous precursors in such architectures would simultaneously address scalability and sustainability requirements.

**Engineering scale-up and techno-economic validation**. Bridging the gap between laboratory synthesis and water treatment application requires sustained collaborative effort between materials scientists, environmental engineers, and water utilities. Priority activities include the development of granulation protocols that preserve composite surface chemistry, fixed-bed column studies under hydraulically realistic conditions, and the first systematic techno-economic analyses comparing zeolite-based composite treatment against established advanced treatment technologies. The alignment of such work with the objectives of SDG 6 (Clean Water and Sanitation), SDG 9 (Industry, Innovation and Infrastructure), and SDG 12 (Responsible Consumption and Production) provides both scientific motivation and policy context for the necessary investment in application-oriented research.

## 8. Conclusions

This review has developed a mechanism-oriented, structure–property–performance framework for zeolite-based composite adsorbents applied to organic micropollutant removal from aqueous systems, drawing on a systematic analysis of literature published between 2016 and 2026. The principal conclusions are as follows.

Pristine zeolite frameworks offer structural precision, chemical stability, and ion-exchange capacity, but are intrinsically limited in OMP adsorption scope by their hydrophilic surface character, restricted interaction diversity, and sensitivity to competitive adsorption by water molecules and dissolved organic matter. These limitations are not overcome by Si/Al ratio optimization alone and define the fundamental motivation for composite material engineering.

The three principal composite strategies examined—carbon modification, polymer and surfactant functionalization, and metal oxide incorporation—each address distinct subsets of these limitations through mechanistically different pathways. Carbon-modified composites expand hydrophobic and π–π stacking capacity, polymer and surfactant systems improve processability and introduce additional electrostatic and hydrogen-bonding sites, and metal-oxide composites enable catalytic contaminant mineralization that transcends the inherent limitation of adsorptive capacity saturation. No single composite class resolves all limitations simultaneously, and the rational selection of a composite strategy must be guided by the molecular properties of the target contaminants, the water matrix composition, and the operational constraints of the intended application.

The five interaction mechanisms governing OMP removal—electrostatic attraction, hydrophobic partitioning, π–π stacking, hydrogen bonding, and pore confinement—operate simultaneously and interdependently, with their relative contributions shifting as a function of contaminant speciation, solution pH, and water matrix composition. This mechanistic complexity implies that adsorption performance is not an intrinsic material property but an emergent outcome of the interplay between material, contaminant, and environment—a conclusion with direct implications for performance evaluation methodology.

Water matrix effects—NOM competition and pore blocking, electrostatic shielding by inorganic ions, and pH-driven speciation changes—consistently reduce adsorption performance relative to idealized laboratory conditions, frequently by factors of two to five. The systematic overestimation of achievable performance through single-solute, ultrapure-water evaluation represents the most significant methodological limitation of the current literature and the most consequential barrier to credible technology assessment.

Regeneration, long-term structural stability, metal leaching, and techno-economic viability remain critically under characterized across all composite classes. The transition from proof-of-concept laboratory materials to deployable water treatment technologies requires coordinated progress in standardized performance evaluation, cyclic stability assessment, granulation and process engineering, and full-scale techno-economic analysis.

The advancement of zeolite-based composite nanomaterials for water purification is directly aligned with the United Nations Sustainable Development Goals, most critically SDG 6 (Clean Water and Sanitation), SDG 9 (Industry, Innovation and Infrastructure), and SDG 12 (Responsible Consumption and Production). Realizing the potential of these materials to contribute to sustainable water management at scale requires a deliberate and coordinated shift from proof-of-concept synthesis toward application-oriented, interdisciplinary research that bridges materials science, environmental engineering, and water treatment practice.

## Figures and Tables

**Table 3:** Representative polymer- and surfactant-modified zeolite composites for contaminant removal from water.

No.	Composite Material/Format	Zeolite Type	Modifier (Polymer/Surfactant)	Target Contaminants	Key Mechanistic Insight	Reference
1	Surfactant-modified zeolite (SMZ)	Natural zeolite (clinoptilolite-rich)	Cationic surfactant layer	Organic contaminants and hydrophobic organics	Formation of an organophilic surface phase enabling hydrophobic partitioning and electrostatic interactions	[7]
2	Cross-linked composite beads	Zeolite nanoparticles	PVA–alginate matrix	Organic and inorganic contaminants	Bead configuration improves sorbent handling, enables column operation, and facilitates sorbent recovery	[56]
3	Structurally modified zeolite adsorbent	NaY zeolite	Framework modification	Diclofenac sodium (pharmaceutical)	Structural tuning of pore environment improves adsorption of pharmaceutical micropollutants	[57]

**Table 4:** Representative metal-oxide-functionalised zeolite composites for OMP removal from aqueous systems.

No.	Composite Material	Target Contaminant	Process	Removal Efficiency	Reference
1	TiO_2_/Zeolite Y	Diclofenac	Photocatalysis	92 ± 3% (range: 90–95%)	[68]
2	TiO_2_/ZSM-5	Carbamazepine	Photocatalysis	88 ± 4% (range: 84–92%)	[76,77]
3	TiO_2_/Clinoptilolite	Pharmaceutical mixtures	Photocatalysis	91 ± 4% (range: >90%)	[64,78]
4	ZnO/Zeolite A	Atenolol, beta-blockers	Photocatalysis	88 ± 4% (range: 85–92%)	[64]
5	ZnO/ZSM-5	Ibuprofen	Photocatalysis	85 ± 3% (range: 82–88%)	[64]
6	Fe_3_O_4_/Zeolite (magnetic)	Ibuprofen	Adsorption–oxidation	87 ± 4% (range: 83–90%)	[65]
7	Fe-ZSM-5	Paracetamol	Fenton oxidation	90 ± 3% (range: 87–93%)	[79]
8	Fe_2_O_3_/Zeolite	Sulfamethoxazole	Photo-Fenton	87 ± 3% (range: 84–90%)	[80]
9	Fe-Zeolite catalyst	Diclofenac	Fenton oxidation	89 ± 4% (range: 85–93%)	[40]
10	CuO/Zeolite Y	Phenolic compounds	Catalytic oxidation	82 ± 4% (range: 78–85%)	[71]
11	CuO/ZSM-5	Phenol	Catalytic oxidation	86 ± 4% (range: 82–90%)	[70]
12	MnO_2_/Zeolite NaY	Dye pollutants	Catalytic oxidation	85 ± 5% (range: 80–90%)	[73]
13	MnO_2_/ZSM-5	Methylene blue	Catalytic oxidation	90 ± 5% (range: 85–95%)	[81]
14	Fe_3_O_4_–TiO_2_/Zeolite	Mixed pharmaceuticals	Photocatalysis	92 ± 3% (range: >90%)	[64]
15	Ag/TiO_2_–Zeolite composite	Pharmaceutical residues	Photocatalysis	93 ± 3% (range: 90–96%)	[82]

Note: Removal efficiency values represent midpoint estimates derived from reported ranges in cited primary studies; ±SD values reflect the spread of reported results across experimental replicates and conditions as documented in the source literature. Where primary studies reported single values without replicates, ±SD is estimated from the reported experimental range. All values obtained under laboratory conditions (single-solute, deionised/ultrapure water, contaminant concentrations of 5–50 mg L^−1^). Performance under environmentally representative conditions (ng L^−1^ to µg L^−1^, real water matrix) may differ substantially.

**Table 5:** Influence of water matrix and operational parameters on OMP adsorption by zeolite-based composite sorbents, with mechanistic interpretation and representative literature examples.

Parameter	Primary Mechanism	Direction of Effect	Composite Type Most Affected	Representative Example	Reference
pH (acidic → neutral)	Contaminant speciation; sorbent surface charge	Variable; typically enhances cationic OMP uptake, reduces anionic OMP uptake on unmodified zeolite	All composite types	Diclofenac adsorption on TiO_2_/zeolite decreases above pH 6 as TiO_2_ surface deprotonates	[86,87]
pH (neutral → alkaline)	Progressive deprotonation of metal oxide surface sites	Reduces electrostatic uptake of anionic OMPs on metal-oxide composites	Metal-oxide–zeolite composites	Ibuprofen removal on Fe-zeolite reduced ~40% between pH 6 and pH 9	[86]
NOM (competitive adsorption)	Displacement of OMP from hydrophobic sites by humic fractions; pore blocking	Reduces adsorption capacity 30–70%	Carbon–zeolite composites most affected	Carbamazepine capacity on AC–zeolite reduced ~50% in presence of 10 mg L^−1^ humic acid	[83,85]
Ionic strength (monovalent ions)	Compression of electrical double layer; suppression of electrostatic interactions	Reduces electrostatic OMP uptake by 15–40% at 10–50 mM	All composite types with electrostatic mechanism	Na^+^ and K^+^ at realistic ionic strength suppress cationic pharmaceutical uptake on clinoptilolite	[48]
Divalent cations (Ca^2+^, Mg^2+^)	Cation bridging between negatively charged surface and anionic OMP	May enhance anionic OMP adsorption via bridging; also competes for ion-exchange sites	Unmodified zeolite; metal-oxide composites	Ca^2+^ presence enhanced diclofenac uptake on zeolite relative to equivalent Na^+^ ionic strength	[84]
Temperature (increase)	Influences thermodynamics (exo/endothermic) and diffusion kinetics	Higher T improves kinetics but may reduce equilibrium capacity for exothermic adsorption	All composite types	Temperature-dependent capacity changes of 10–25% reported across pharmaceutical–zeolite systems	[88]

## Data Availability

The original contributions presented in this study are included in the article.

## Abbreviations

**BEA**	Beta zeolite framework topology
**BET**	Brunauer–Emmett–Teller (surface area analysis method)
**CEC**	Cation exchange capacity
**CNT**	Carbon nanotube
**CPC**	Cetylpyridinium chloride
**DOC**	Dissolved organic carbon
**DOM**	Dissolved organic matter
**EDC**	Endocrine-disrupting compound
**FAU**	Faujasite zeolite framework topology
**GO**	Graphene oxide
**HDTMA**	Hexadecyltrimethylammonium
**HEU**	Heulandite zeolite framework topology
**MFI**	Mobile five zeolite framework topology (ZSM-5, silicalite-1)
**MOR**	Mordenite zeolite framework topology
**NOM**	Natural organic matter
**OMP**	Organic micropollutant
**PFAS**	Per- and polyfluoroalkyl substances
**PFOA**	Perfluorooctanoic acid
**PFOS**	Perfluorooctane sulfonate
**PPCP**	Pharmaceutical and personal care product
**PVA**	Poly(vinyl alcohol)
**rGO**	Reduced graphene oxide
**SDG**	Sustainable Development Goal
**SMZ**	Surfactant-modified zeolite
